# Chitosan: A Sustainable Material for Multifarious Applications

**DOI:** 10.3390/polym14122335

**Published:** 2022-06-09

**Authors:** Abdul Zubar Hameed, Sakthivel Aravind Raj, Jayakrishna Kandasamy, Majed Abubakr Baghdadi, Muhammad Atif Shahzad

**Affiliations:** 1 Department of Industrial Engineering, Faculty of Engineering, King Abdulaziz University, P.O. Box 80204, Jeddah 21589, Saudi Arabia; aahameed@kau.edu.sa (A.Z.H.); mbaghdadi@kau.edu.sa (M.A.B.); mmoshtaq@kau.edu.sa (M.A.S.); 2 Department of Manufacturing Engineering, School of Mechanical Engineering (SMEC), Vellore Institute of Technology, Vellore 632014, India; mail2jaikrish@gmail.com

**Keywords:** chitosan, antibiotic, Gram-positive and -negative bacteria, drug delivery, biodegradable, heavy metal removal, COVID-19, wound healing

## Abstract

Due to the versatility of its features and capabilities, chitosan generated from marine crustacean waste is gaining importance and appeal in a wide variety of applications. It was initially used in pharmaceutical and medical applications due to its antibacterial, biocompatible, and biodegradable properties. However, as the demand for innovative materials with environmentally benign properties has increased, the application range of chitosan has expanded, and it is now used in a variety of everyday applications. The most exciting aspect of the chitosan is its bactericidal properties against pathogens, which are prevalent in contaminated water and cause a variety of human ailments. Apart from antimicrobial and water filtration applications, chitosan is used in dentistry, in water filtration membranes to remove metal ions and some heavy metals from industrial effluents, in microbial fuel cell membranes, and in agriculture to maintain moisture in fruits and leaves. It is also used in skin care products and cosmetics as a moisturizer, in conjunction with fertilizer to boost plant immunity, and as a bi-adhesive for bonding woods and metals. As it has the capacity to increase the life span of food items and raw meat, it is an unavoidable component in food packing and preservation. The numerous applications of chitosan are reviewed in this brief study, as well as the approaches used to incorporate chitosan alongside traditional materials and its effect on the outputs.

## 1. Introduction

**Figure 1:**
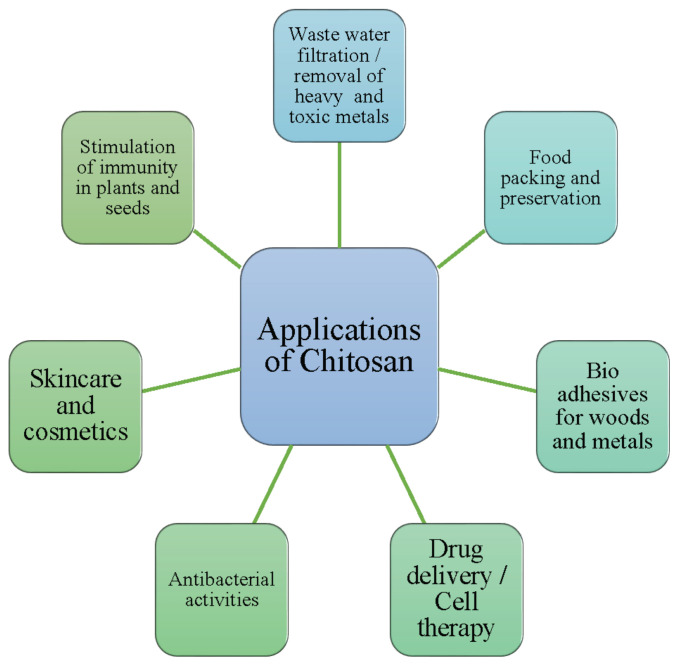
Various applications of chitosan.

Chitosan is a sugar found mostly in the shells of crustaceans such as crabs, shrimps and lobsters. It is a drug that is used in the pharmaceutical sector. Chitosan, a fibrous substance, may help the body absorb less fat and cholesterol from the foods we eat. It assists in the production of blood clots when applied to wounds. Chitosan is utilized in a variety of applications, including contaminated drinking water, which has a significant impact on human health, and experts are working to develop the best disinfectant to address this issue. Nanocomposites containing chitosan and carbon nanotubes have been identified as viable alternatives to conventional disinfection methods [1]. Nanomaterial-based membranes were discovered to be a sustainable method of treating waste fluids. Membranes and reactors treated with chitosan demonstrated increased resistance to key contaminants and decreased membrane fouling [2]. Chitin and chitosan are formed from fish debris such as heads, tails, skins, scales, and shells, which are unfavorable to humans and the environment [3]. The presence of heavy metals in water is greater, and if consumed in this state, will have a variety of adverse consequences on human health. Chitosan is an excellent absorber of heavy metal ions and is utilized in industry for waste water treatment in fixed-bed column designs [4]. Chitosan makes a greater contribution to biomedical research by acting as an antibiotic, antioxidant, and drug delivery agent. Due to the additional benefits, such as the non-toxicity and practicality, chitosan is well suited for medical purposes. Due to the deficiencies of chitosan in terms of cell adhesion and biosignaling, peptide chitosan has been developed with enhanced cell adhesion and biosignaling capabilities and is being employed in cell therapy, drug delivery, and as an antimicrobial [5]. Due to decreasing petroleum resources and the environmental damage caused by petroleum products, biomaterials are garnering increased attention. Chitosan is used in a range of industries, including water treatment, medicine, fisheries, and cosmetics; the chemical and packaging industries; as well as food and agriculture. Additionally, it is utilized in the exploration, extraction, refining, and treatment of waste water for petroleum products. Chitosan must undergo particular chemical alterations in order to be used in petroleum field applications [6]. Vacuum-aided filtering, freeze-casting, and biomimetic mineralization are used to make chitosan–calcium phosphate composites. It can be applicable in drug administration, bone implants, wound healing, dental implants, and waste water filtration from heavy and organic metals [7]. Chitosan also possesses antiviral properties and can be utilized as an adjuvant in vaccines against SARS and COVID viruses [8]. Chitosan is a derivative of chitin that exhibits antifungal, biodegradability, biocompatibility, mucoadhesion, and antibacterial properties. It is used in dental and bone implants [9]. Membrane development for water purification is a significant area of research in which a number of researchers are involved. Natural and manmade polymers derived from chitosan have demonstrated their ability to purify water. Chitosan-based nanocomposites are favored for water purification applications because of their low cost, non-toxic nature, biodegradability, and biocompatibility. Chitosan is capable of eliminating heavy metals, dyes, and other hazardous contaminants from water during the purification process [10]. Chitosan is a polysaccharide found naturally in marine crustaceans. Chitosan can also be applied following molecular and chemical changes, including in biomedicine, the pharmaceutical sector, agriculture, gene research, drug delivery, imaging, wound healing, and tissue engineering [11]. The various applications of chitosan are illustrated in Figure 1. The various sources of chitosan, the methods of extraction, the chemical modifications required, the incorporation of chitosan into composites for various applications, and the use of chitosan in various fields are discussed succinctly in this review for the benefit of researchers who work on the application of chitosan for their specific purposes. Table 1 indicates the biological and physicochemical properties of chitosan based on the degree of N-acetylation and the molecular weight.

## 2. Chemical Properties and Processing Technologies of Chitosan Based Materials

### 2.1. Physicochemical and Biological Activities

**Figure 2:**
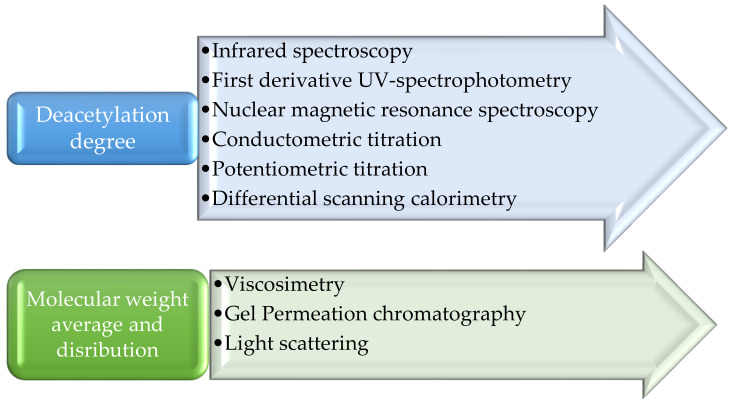
Determination methods followed for Deacetylation degree and Molecular weight distribution and average.

The deacetylation process is the hydrolysis process of acetamide groups in chitin when strong NaOH solution reacts at temperatures of 100 °C and above, producing the amino groups of the new compound known as chitosan. The formed amino groups in the chitosan decide its biological properties. The deacetylation degree range of 70–85% in chitosan means it can be partly dissolved in water, and above 95 to 100% is the ultrahigh DD range of chitosan, which is a challenging task to achieve. The DD and Mw distributions and average Mw can be determined using the methods mentioned in Figure 2 [13]. Table 2 shows the biological and chemical properties of chitosan.

Table 3 indicates the physicochemical and biological properties of chitosan with respect to its DA and Mw. The direct and indirect proportionality levels of Mw and DA with the properties of chitosan can be easily understood and can be applied based on the required applications [14]. Crystallinity and hydrophobicity characteristics are influenced by the acetylation degree. Reacetylated chitosan is applied as a coating in cardboard to improve the mechanical properties. Chitosan with a 2% degree of acetylation showed better water and mechanical resistance. This indicates that the molecular groups are well distributed, which increases the hydrophobicity of the polymers [15].

**Figure 3:**
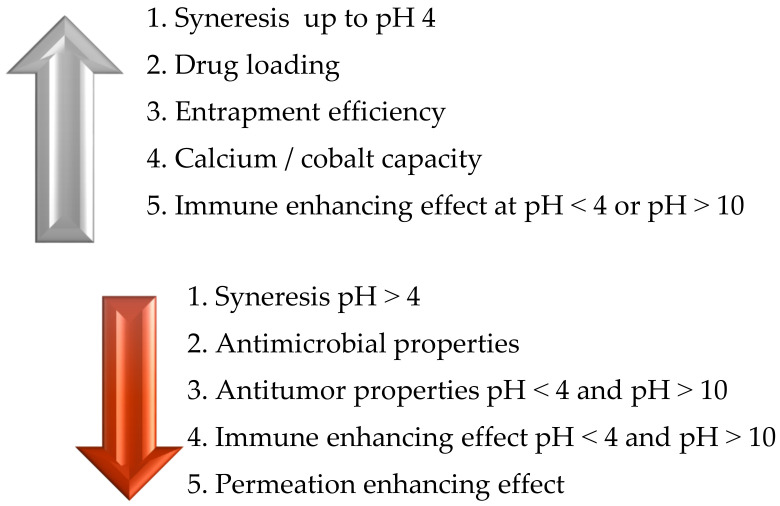
Influence of pH on the biological and physicochemical characteristics of chitosan.

**Figure 4:**
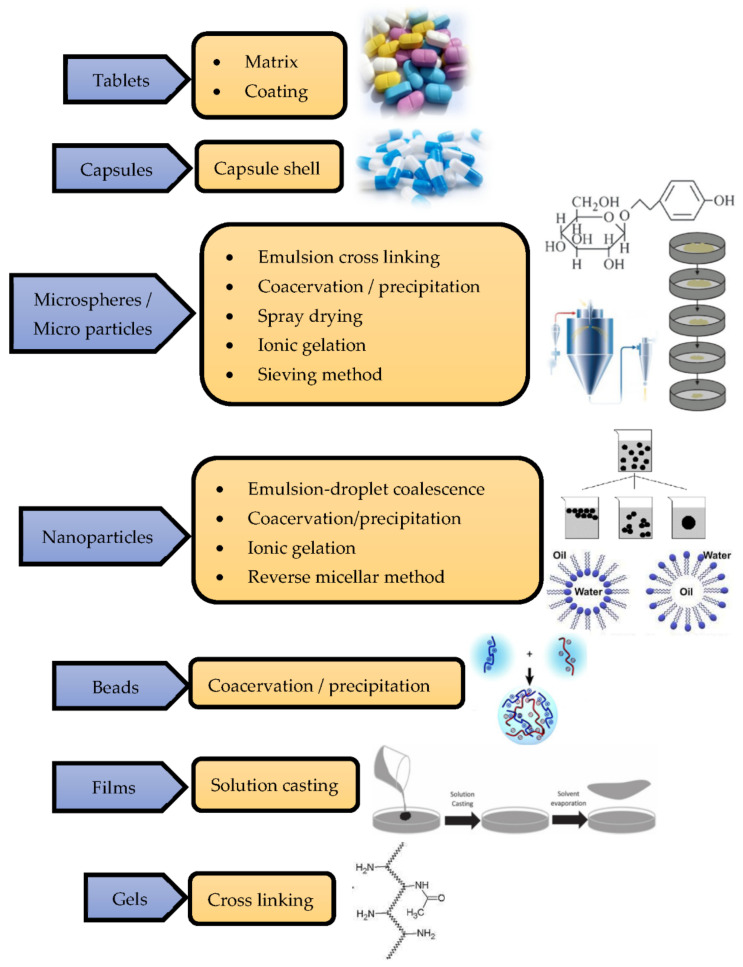
Processing technologies for making products using chitosan.

If the pH value increases, the drug loading, entrapment efficiency, and immune enhancement effects are enhanced, while if the pH value decreases, the antimicrobial properties, antitumor properties, and permeation effect are reduced. The values at which the property changes occur are also mentioned in Figure 3. Chitosan can be embedded, encapsulated, mixed, precipitated, spray-dried, emulsified, crosslinked, and cast into various products such as tablets, capsules, and nano- and microparticles, and can be formed into beads, films, and gels. The processing technologies through which the chitosan can be incorporated into other materials are described in Figure 4 [16].

### 2.2. Process Technologies for Producing Chitosan Composites

**Figure 5:**
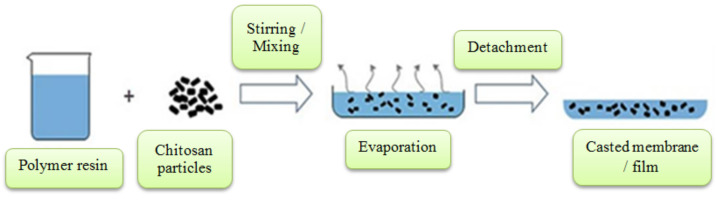
Solvent casting for film and membrane manufacture.

Membranes and films that can be used in air and water filtration processes can be made using the solvent evaporation process. Solvent evaporation is a simple three step process in which the polymer resin is mixed with nano- or micron-sized chitosan fillers; sometimes fibers may also be reinforced to enhance the mechanical properties. The film and cast membrane manufacture process is described in Figure 5. The mixed solution is then poured into a glass container and heated to initiate the evaporation process. After evaporation, the cast membrane or film can be taken out from the container [17].

**Figure 6:**
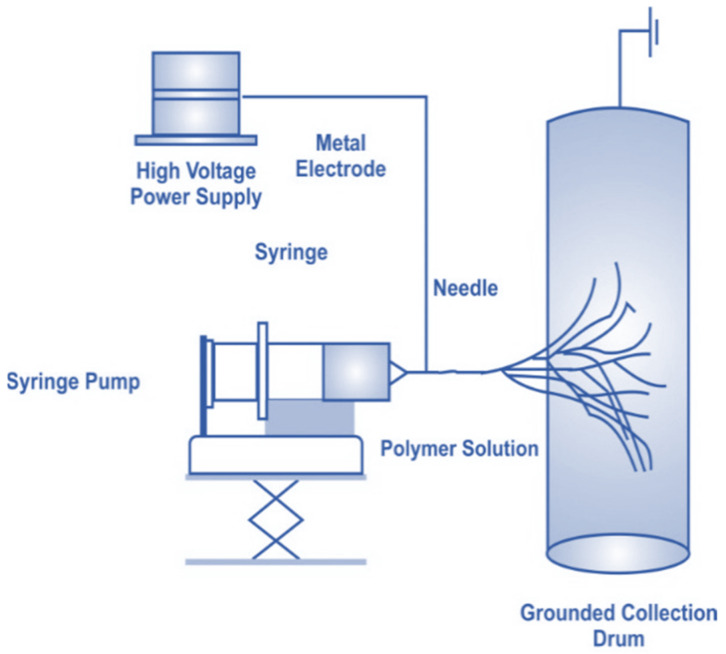
Electrospinning process with rotating mandrel [18].

**Figure 7:**
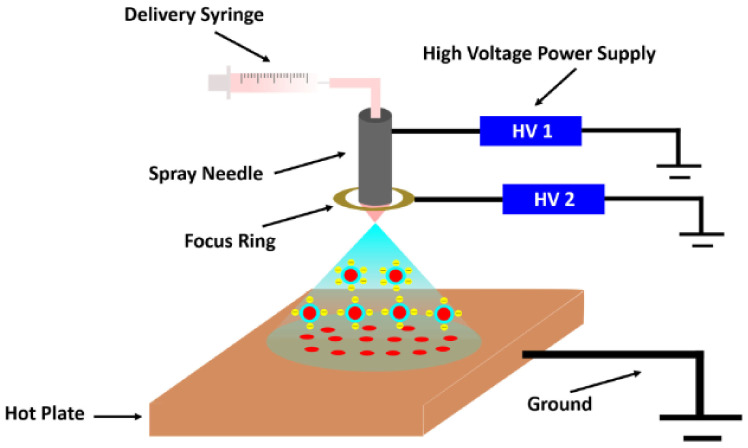
Deposition of chitosan via electrospraying method. Reprinted from [19]. Copyright 2022 with permission from the American Chemical Society.

The process of electrospinning and the equipment required are shown in Figure 6. The major differences between conventional fibers and electrospun fibers are their diameter and surface area. The method used for depositing nanochitosan on oppositely charged substrates is shown in Figure 7. The electrospun fibers have a larger surface area and smaller diameter. The polymer solution is subjected to differences in potential generated between the spinneret and collector. Due to the electric filling, the pendant-like droplets are converted into jets, and at a critical value the repulsion of the electricity exceeds the tension offered by the solution on its surface. Due to this phenomenon, the extruded polymer solution is subjected to rapid whipping, which is unstable, while the evaporation leads to nanofibers forming on the collector [18].

**Figure 8:**
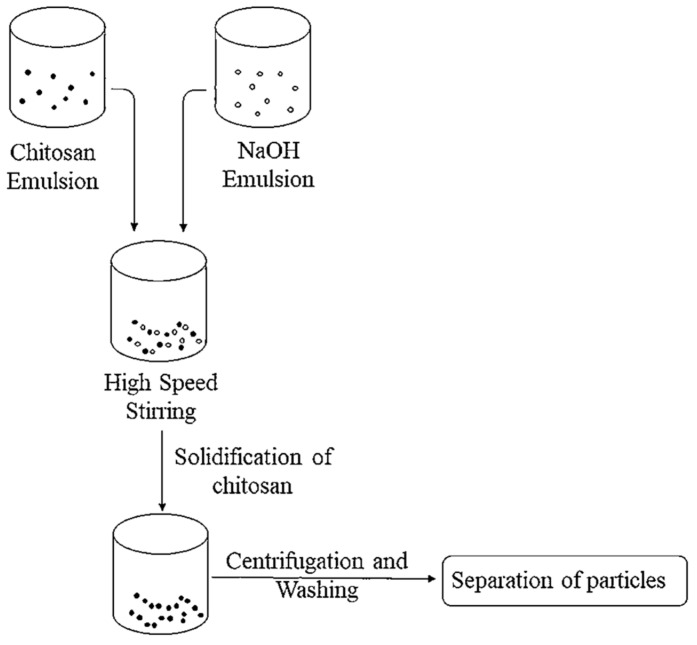
Emulsion droplet coalescence method used to produce chitosan nanoparticles [21].

In this process, the chitosan particles are mixed with a polymer solution and kept in a dispersion needle. A high voltage is supplied to the solution in the needle. Due to the identical charge the droplets repel each other, then due to instability at the needle tip the droplets start dispersing into micron-sized particles and are deposited on oppositely charged surfaces, while the solvent is evaporated rapidly [20]. The method of producing chitosan nanoparticles through emulsion droplet coalescence is described in Figure 8.

**Figure 9:**
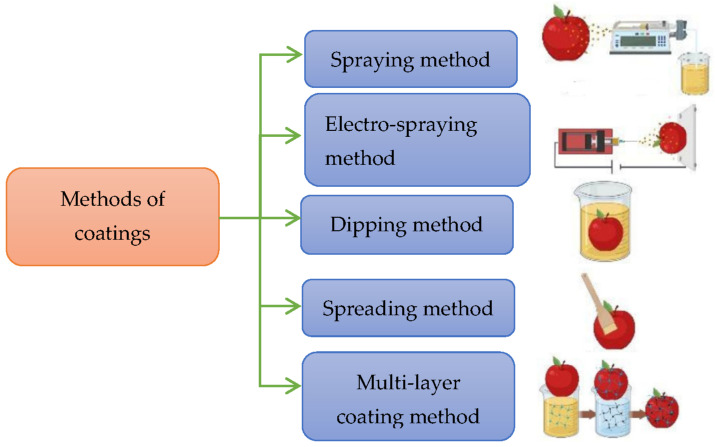
Methods used for coating fruits and vegetables [22].

In Figure 9, the different methods for applying biodegradable coatings on fruits and vegetables are listed. The best alternatives to wax coatings were found, which were chemically formulated. These coatings prevented microbial loading on fruits by resisting against oxidation and reduction. By minimizing the vapor dissipation, decay, and ripening caused by bacteria, the physiological and microbial deterioration is reduced and the shelf lives of the fruits can be extended [22].

**Figure 10:**
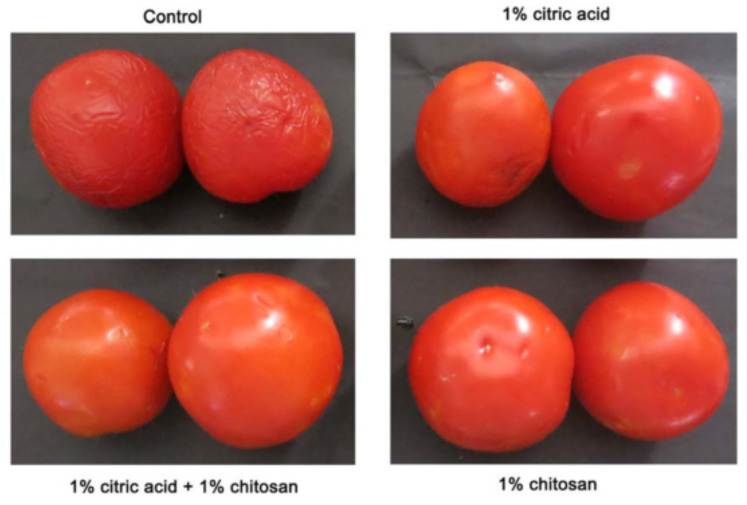
Conditions of tomato samples coated with chitosan and citric acid after 15 days at 28 °C [23].

Post-harvest, the fruits and vegetables are coated with chitosan to maintain their quality. Pure chitosan or a combination of chitosan with citric acid is used for coating. The conditions of post-harvested tomatoes after 15 days with different coating levels and elements are shown in Figure 10. Tomatoes coated with chitosan in combination with citric acid showed less undesirable changes, less weight loss, and less tissue damage, confirming the usage of chitosan as a preservative coating on fruits and vegetables [23]. It is safe to use chitosan similarly to salts as a coating in fruits, as it is non-toxic when consumed by humans.

## 3. Major Applications of Chitosan

### 3.1. Water and Air Filtration

**Figure 11:**
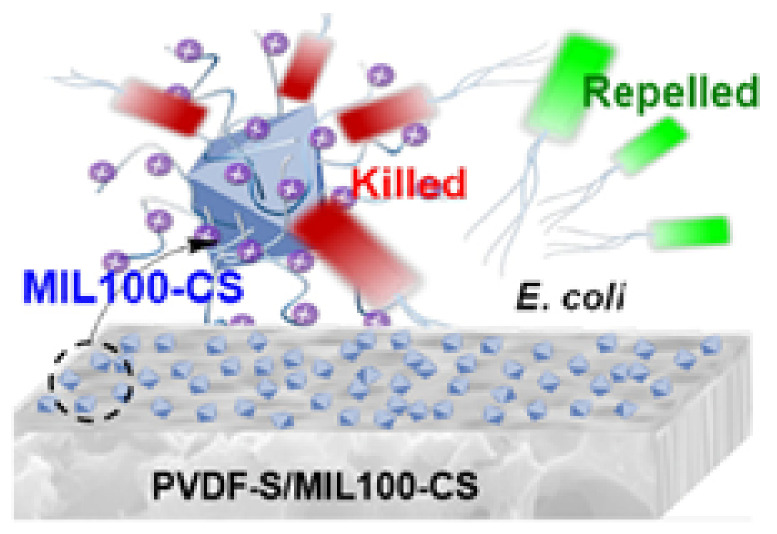
Antibacterial inhibition activity illustration of composite made of PVDF-S/MIL100-CS [29].

**Figure 12:**
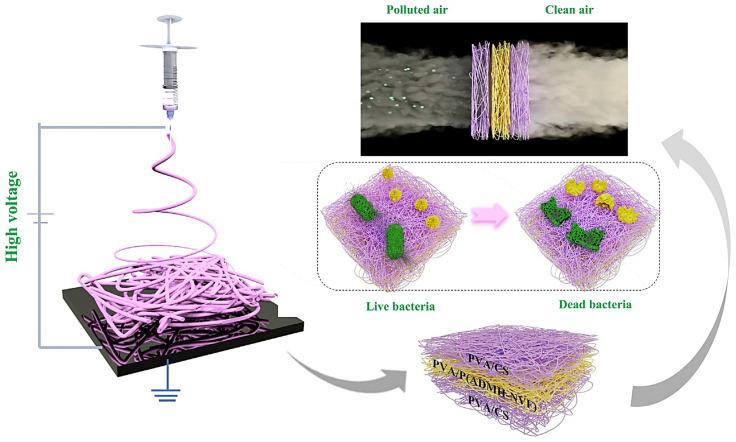
Electrospinning process used to fabricate multilayer air filters. Reprinted with permission from [32]. Copyright 2020 with permission from Elsevier.

By and large, waste water is contaminated with bacteria and microorganisms that cause a variety of ailments. It was discovered that CS with a low Mw inhibits the growth of Gram-positive bacteria, whereas chitosan with a high molecular weight inhibits bacteria. Additionally, it has been demonstrated that doping chitosan with nanoparticles enhances its antibacterial characteristics, and that when coupled with silver nanoparticles, a low concentration of chitosan is sufficient to control bacteria that cause water contamination [24]. Chitosan–cobalt–silica nanocomposites were prepared and utilized for dye absorption and water purification. Additionally, this combination was investigated against bacteria, and the results indicated that it exhibited significant bioactivity toward them [25]. Chitosan composites can be used in special filters in the textile industry, as it has better color absorption properties, along with the ability for metal ion absorption. The PVA-co-PE nanofiber membrane was used to clean waste water and was shown to have a higher water flux and retention rate for nanoparticles and bacterial cells. The membrane’s bacteria inactivation rate was also increased from 97.8 to 99.5% against pathogens, and the membranes were capable of being employed for extended periods of time with high stability and efficiency [26]. The filtration of wastewater and industrial effluents using membrane filters has the significant disadvantage of membrane fouling, which prompted the creation of chitosan-based antifouling membranes. Through spinning, chitosan and silver NP’s were incorporated into membranes of hollow fibers. The chitosan- and silver-chitosan-based membranes showed superior performance with the highest dye rejection rates, and were determined to be the most suitable for treating industrial effluents without fouling the membrane [27]. To create a multifunctional composite, chitosan, polyethyleneimine, graphene oxide, and glutaraldehyde were combined and coated with membranes capable of removing both positively and negatively charged heavy ions. The glass microfiber filter was chosen and was found to be effective in removing Cr (VI) and Cu (II), demonstrating that this coated membrane may be utilized to remove both positive and negative ions from water [28]. Water flux reduction in membranes used for water treatment is a critical issue owing to the associated bacterial development. To create a PVDF-S/MIL100-CS composite, a recently discovered approach termed solvent-assisted nanoparticle embedding was applied. The new production procedure spread the fillers over an open surface, imparting a hydrophilic quality to the surface. The antibacterial activities of the PVDF-S/MIL100-CS composite are mentioned in Figure 11. The results suggested increases in antibacterial activity and resistance to biofouling, which was validated by a live/dead test for antibacterial activity [29]. The fluoride pollution of groundwater appears to be a significant issue, and numerous experts are striving to develop a cost-effective remedy. A mixed matrix membrane (MMM) based on cellulose acetate was created using the phase inversion approach, together with the use of mixed metal oxide nanoparticles as nanofillers. The results of the tests indicated that the MMM was less susceptible to attack by microbes, and it was discovered that the fluoride ion was rejected due to adsorption, while the membrane surface exhibited electrostatic repulsion, enhancing the defluorination effectiveness [30]. The MWNT was disseminated in an aqueous solution containing varying quantities of chitosan, and the membrane was formed. The bucky paper membranes have excellent mechanical qualities, and their zeta potential improves as the amount of added chitosan increases. Additionally, it was observed that the bucky paper membrane modified with MWNT exhibited improved salt rejection capabilities and smaller interior pores [31]. Bacterial dispersal in the air, combined with particulate matter pollution, is increasing daily, posing a threat to human health. Multilayer membranes with antibacterial properties and excellent air filtration efficiency need to be developed. Sequential electrospinning was used to generate PVA/chitosan membranes with N-halamine that displayed high filtration efficiency and tensile strength in the filtration test. The process of producing multilayer air filters through electrospinning is mentioned in Figure 12 [32]. More efficient water filtration systems are required to address national and global water scarcity challenges. Researchers are focusing their attention on the development of low-cost membranes. PAN membranes were enhanced with nanoparticles of zinc oxide and chitosan to improve the water filtration, mechanical, and antibacterial qualities. It was evident from the results that the created composite membranes possessed excellent antibacterial and self-cleaning qualities [33]. Water filters capable of removing metal ions were created by electrospinning nylon/chitosan fibers. These fibers were tested against lead nitrate and sodium chloride, and the results demonstrated that the membrane was capable of removing metal ions and bacteria from an aqueous solution to a concentration of up to 96% [34]. Numerous contagious diseases are conveyed via air, and numerous concerns have been raised about aerosols and bioaerosols. Electrospinning is used to create polyurethane/chitosan nanofibers, meaning various parameters such as the diameter are changed, the effects of which are examined. The nanofibers demonstrated superior performance when used in filtration processes in industry and in equipment used for respiratory purposes [35]. Superhydrophobic poly (methylmethacrylate)/polydimethylsiloxane fibers with a capture efficiency of 98.23% are used to catch particle matter. On a window screen, continuous particle removal has been proven to filter the particles [36]. Water pathogens and bacteria form biofilms and lead to biofouling, which continue to be significant concerns in many locations. Membranes composed of chitosan, PEG, MWCNT, and iodine were made in three phases. The inclusion of iodine increased the hydrophilicity, porosity, and performance of the membranes. The reduced iodine concentrations killed 99.2 and 100% of *E. coli* and *S. aureus* bacteria, respectively [37].

**Figure 13:**
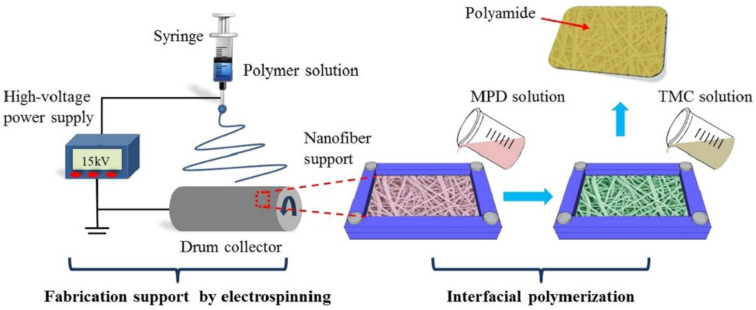
Electrospinning process used to fabricate multilayer water filters [38].

**Figure 14:**
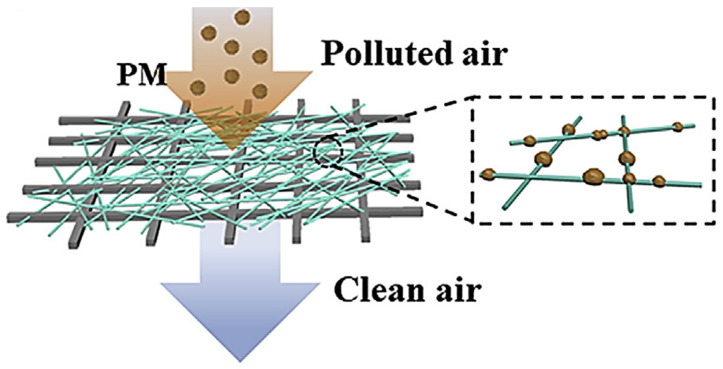
Illustration of fibrous membrane in which the pollutants of air are filtered [36].

Removal of dyes and organic pollutants is a difficult task that can be made possible through incorporating chitosan along with multilayer composite membranes. The production of water filters through the electrospinning process is described in Figure 13. Polyacrylonitrile nanofibers produced via the electrospinning process and supported by polyamide membranes are used to filter waste water and to remove tetracycline from it. These fibers have been laminated and tested and found to be more effective in tetracycline removal [38]. In Figure 14, the filtration process of air pollutants through a fibrous membrane has been illustrated.

Regarding the unique antibacterial mechanism of chitosan-reinforced PVDF-S/MIL100 composites, the negatively charged *E. coli* combine with oppositely charged chitosan. Some *E. coli* bacteria were repelled from the composite surface due to its hydrophillic nature. This property of killing the bacteria or repelling the bacteria from the filter surface is most needed for the air and water filters, meaning the addition of chitosan to the filter membranes is very much needed and effective. When compared to single-layer membranes used for filtering air and water, multilayer membranes have been found to be more effective in filtering the bacteria and other pollutants. The filtration efficiency increases when the number of layers increases. Electrospinning has been found to be the most suitable method for manufacturing filter membranes for both air and water purification purposes. Additionally, the usage of chitosan in the filter membranes has added benefits, such as making the material non-toxic and biodegradable, because when used along with water filters, it may mix with water or be washed away along with the water flow, meaning humans may intake chitosan mixed with water. In such cases, chitosan has no toxicity and is safe. After a certain time period based on the usage of water the filters need to be replaced, and in such cases the used filters may cause pollution issues, which should be avoided as chitosan is biodegradable.

### 3.2. Metal Removal from Water

Industrial effluents have a high concentration of heavy metals that can cause major diseases and organ damage, meaning they must be separated from drinking water. Metal ions contained in contaminated water are also harmful to health, and their removal from the water is a difficult process that is accomplished by adding chitosan to the filtration membrane. Separating a catalyst from a reaction media is a difficult task. This was accomplished by coating the high surface area of the filter paper with chitosan to increase its affinity for metal ion absorption. The filter paper is composed of cellulose microfibers that act as a support for the catalyst. The catalyst can be recovered and utilized for subsequent chemical reactions using this approach [39]. Chitosan is used to cover cellulose filter paper and is used to adsorb Ni^2+^ ions from aqueous solutions. To transform the adsorbed ions into nanoparticles, they are treated with a NaBH4 solution. FESEM and EDAX are used to characterize the transformed nanoparticles. It has been demonstrated that filter paper containing Ni and CS can be used to detect and catalyze other nanoparticles. 

**Figure 15:**
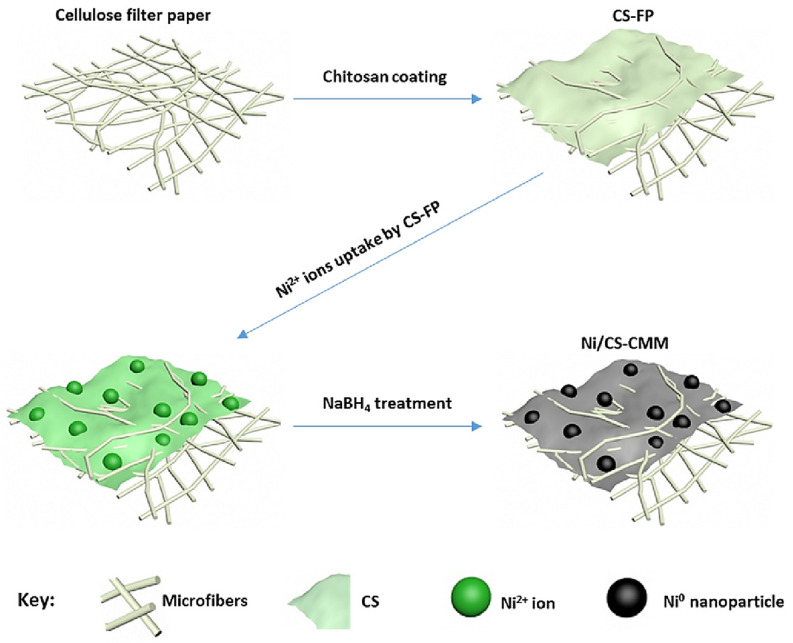
Steps in the preparation of Ni/CS filter paper. Reprinted from [40]. Copyright 2016 with permission from Elsevier.

**Figure 16:**
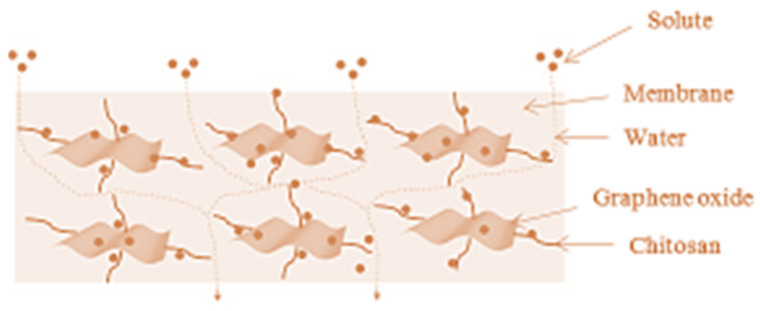
Graphene oxide–chitosan dispersion in water filtration membrane and its feed transport route. Reprinted from [42]. Copyright 2018 with permission from Elsevier.

The filter papers containing chitosan and nickel are described in Figure 15. The production process starts with treating the filter paper with a chitosan solution. Then, the paper is dried and kept in a 2 M NiCl_2_.6H_2_O solution for absorption of Ni^2+^ ions due to the presence of chitosan chains [40]. Here, carboxylated chitosan was deposited onto a membrane and treated with an aqueous copper (II) chloride solution; copper nanoparticles and this thin film membrane were then treated with glutaraldehyde. For 90 days of immersion in water, thin film membranes containing carboxylated chitosan treated with copper (II) chloride demonstrated greater than 99% efficacy against protein fouling. It was demonstrated that chemically modified chitosan acts as an antiprotein fouling agent with increased hydrophilicity [41]. By integrating chitosan and graphene oxide into a polyehtersulfone (PES) membrane, chromium removal and antifouling capabilities can be achieved. The modified membrane demonstrated increased hydrophilicity, a smoother surface, and increased water flux. Additionally, graphene oxide and chitosan-filled membranes exhibit improved antifouling properties. Figure 16 shows a schematic representation of the water filtration process through graphene oxide–chitosan membranes [42]. 

**Figure 17:**
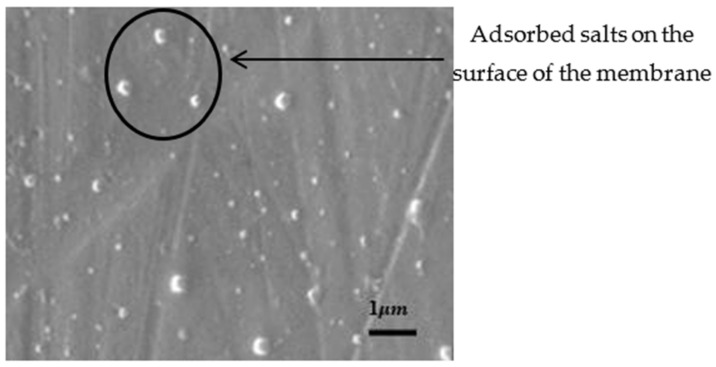
SEM image of membrane with lower graphene–chitosan levels. Reprinted from [42]. Copyright 2018 with permission from Elsevier.

The SEM images shown in Figure 17 indicate the presence of adsorbed particles, which is proof of the adsorption characteristics of filtration membranes reinforced with chitosan. This image was taken after the salt filtration process. The technique of non-solvent-induced phase inversion was utilized to fabricate a thin membrane made of polyvinyl alcohol, chitosan, and montmorillonite clay. Due to its hydrophilic character, this composite membrane demonstrated a higher rejection rate. The heavy metal chromium removal was verified using EDAX and FT-IR measurements [43].

**Figure 18:**
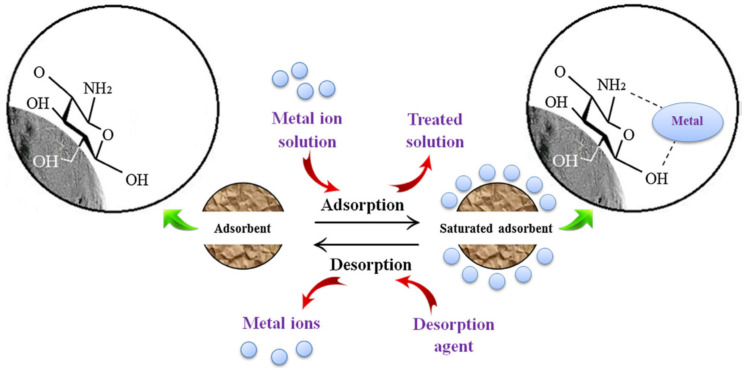
Regeneration process during adsorption. Reprinted from [44]. Copyright 2019 with permission from Elsevier.

Regeneration is an important process in filter membranes to restore the properties of chitosan after the adsorption of heavy metals. Various agents are used for desorption and regeneration processes, such as alkalis, acids, chelating agents, and salts. These agents must also possess certain other properties, such as being non-toxic, biodegradable, and less expensive. Acidic eluents such as nitric acid, hydrochloric acid, phosphoric acid, and sulfuric acids are used as desorption eluents and for regeneration. The regeneration process through which the base properties of the chitosan are regained by using desorption agents is explained schematically in Figure 18 [44].

### 3.3. Antibacterial Activities

**Figure 19:**
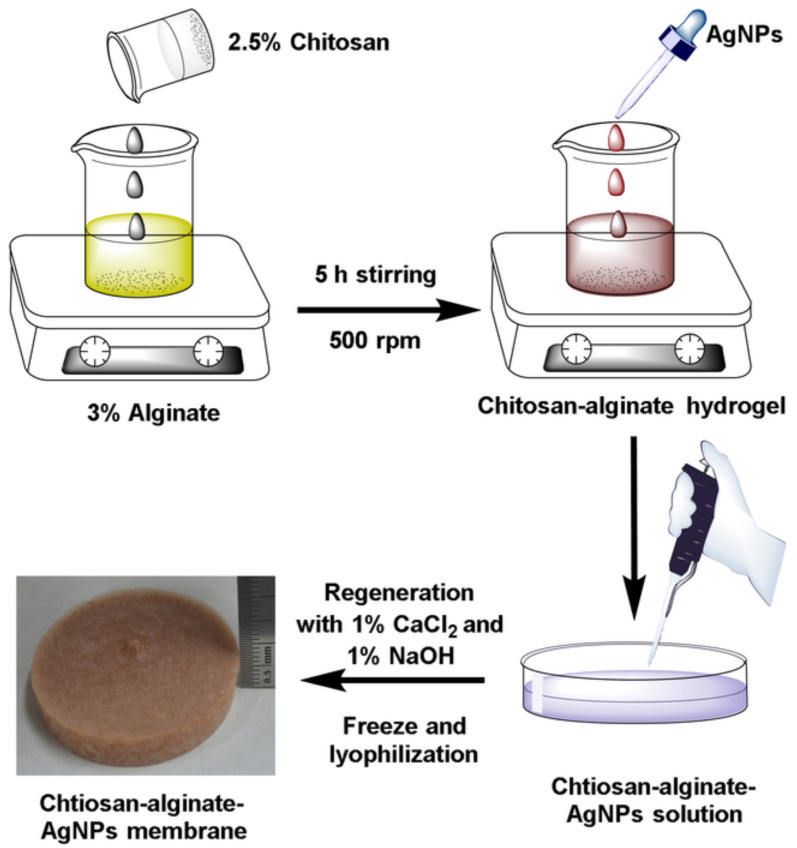
Chitosan–alginate membrane preparation process. Reprinted from [46].Copyright 2017 with permission from Elsevier.

Chitosan possesses unique antibacterial characteristics, particularly against Gram-positive and -negative microorganisms. It can be coated or electrospun with a filter membrane made of conventional material to increase the membrane’s characteristics and performance. The antifouling ability and retention of nanoparticles, as well as the membrane’s hydrophilicity and porosity, can be increased. In aqueous solution, chitosan and silver nanoparticles operate as both stabilizers and reductants. Due to the high stability and monodispersity of chitosan-functionalized silver colloids, it has been evaluated for its antibacterial activity against fungi and bacteria and shown to have a higher bactericidal efficacy against them [45]. The bactericidal actions of chitosan, alginate, and silver nanoparticles were evaluated against *E. coli* and *S. aureus*. Chitosan and alginate are employed to create pores, while silver provides antibacterial action. The chitosan–alginate–silver nanoparticle combination demonstrated superior antibacterial activity against bacteria and demonstrated its potential for usage in the treatment of breast cancer. To prepare a chitosan–alginate membrane, alginate solution is slowly added to a chitosan solution drop-by-drop. A polyelectrolyte complex between chitosan and alginate is formed, which is then dispersed by a high-speed stirrer at 500 rpm. The addition of Ag nanoparticles results in a brownish yellow color. A porous scaffold is obtained via the regeneration of chitosan–alginate–Ag NP’s using sodium hydroxide and calcium chloride. The production of chitosan–alginate membranes and the step-by-step process is explained schematically in Figure 19 [46]. 

**Figure 20:**
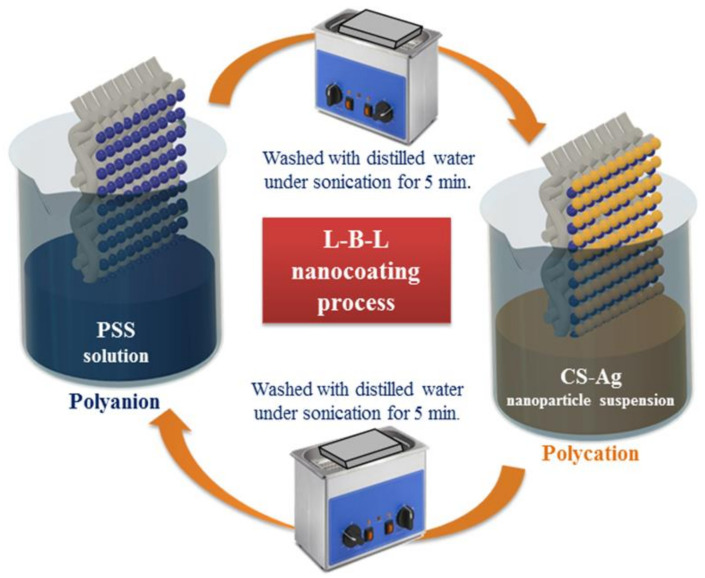
Formation of layers of PSS-CS-Ag on fabric through coating process. Reprinted from [56]. Copyright 2020 with permission from Elsevier.

ZnPc-CS composites were made by dispersing ZnPc in chitosan solution and then immersing the composites in salt solution for metal ion adsorption. The composites were treated with sodium borohydride solution for the conversion of metal ions into nanoparticles. Metal-nanoparticle-loaded composite fibers were synthesized in situ and found to be effective against pathogenic bacteria of *E. coli* and to have the ability to reduce 4-nitrophenol, methyl orange, and Cango red [47]. Quaternized chitosan-embedded membranes made in acetic acid medium have a greater effect on *E. coli* and have 99.95% efficiency [48]. Nitric oxide is a free radical that can be engaged in antibacterial actions such as wound healing. S-nitrosoglutathione was added to pluronic F-127 and chitosan as a nitric oxide donor. The GSNO-PL/CS combination was found to release nitric oxide and was proven to be harmless to Vero mammalian cells [49]. Composite membranes composed of chitosan–collogen, chitosan–collogen–montmorillonite, and chitosan–collogen–organomontmorillonite were investigated for their swelling ratio, moisture permeability, and in vitro degradation ratio properties. It was discovered that they have a higher swelling ratio, excellent moisture permeability, and a lower degradation ratio. To increase the antibacterial activity of the composite membrane, Calicarpa nudiflora was added [50]. *T. portulacifolium* leaf extract was utilized as a reducing agent, and the hybrid composite’s antibacterial efficacy was determined. The mixture demonstrated improved inhibitory activity against microbes such as *S. marcescens* [51]. Antibiotic treatments are carried out using biodegradable and biocompatible polymer-based Nano capsules, also known as hollow nanoparticles. These are self-assembled polysaccharides that are coated with gold nanoparticles to act as a sacrificial matrix layer. Colloidal gold is removed using cyanide-assisted hydrolysis. The combination of chitosan and alginate is an effective antibacterial substance [52]. Chitosan and chitooligosaccharide were added to the cellulose matrix to increase the antibacterial activity, and tests were performed against Gram-positive and Gram-negative bacteria. The results indicated that they have favorable antibacterial activities, and when compared to pure bacterial cellulose (matrix), the BC-CS and BC-COS exhibited low porosity and a dense structure. This BC-COS composite material has excellent suitability for food and medicinal applications [53]. The antimicrobial activity of chitosan nanoparticles against tomato phytopathogens was assessed via the preparation and testing of chitosan nanoparticles. The testing process involves the use of pathogens such as Phytophthora capsici, Colletotrichum gelosporidies, Sclerotinia sclerotiorum, Gibberella fugikuori, and Fusarium oxysporum. Chitosan nanoparticles had a higher inhibitory impact on phytopathogenic bacteria, and both chitosan and chitosan nanoparticles prohibited the development of Erwinia and Xanthomonas [54]. Silver nanoparticles encapsulated in chitosan–silica scaffolds were synthesized using an electrospinning approach, as well as Ag/CS/silica composites. The tests were conducted with and without the inclusion of silver nanoparticles, and the results indicated that the inhibitory effect against bacteria was strengthened, while the addition of silica improved the composite’s mechanical qualities. Biostatic activity was also seen as the diameters varied [55]. To disclose the antibacterial activity of chitosan on fabric made of cotton, a process of layering was used to coat it in its self-assembled form. Through layer-by-layer deposition, silver-loaded chitosan nanoparticles were coated up to 15 bilayers. The fabric displayed effective antibacterial characteristics without compromising its fundamental properties, including its tensile strength, bending stiffness, and air permeability. The base layer in the fabric was formed by dipping it in PSS and PAH aqueous solutions. The body layers were formed after the formation of the base layers by immersing the fabric in 0.6% PSS solution and a nanoparticle suspension consisting of chitosan and Ag. Each immersion was followed by washing with distilled water under sonication. The process can be repeated based on the number of layers required. PSS-CS-Ag layer formation on fabrics is explained in Figure 20 [56]. 

Chitosan–graphite was produced using silica nanoparticles and the bactericidal action towards bacteria was determined after 18 h at 310 K. The production of radical oxygen species occurred as a result of the UV radiation, which caused damage to the bacteria [57]. The effects of nano cerium oxide particles on chitosan films were examined, and antibacterial activity against *E. coli* and *S. aureus* bacteria was discovered. Additionally, this composite film was recommended for use as a coating and packaging material due to its high mechanical strength, flexibility, and antibacterial efficacy [58]. Antimicrobial scaffolds consisting of cuprous oxide nanoparticles and chitosan nanofibers were produced. The Cu_2_O particles became smaller and their shape changed from cubic to irregular as the concentration of CuSO_4_ increased. The composite exhibited increased hydrophilicity and antimicrobial action against both Gram-negative and -positive bacteria [59]. A starch-based film was created and coated with chitosan nanoparticles for antibacterial activity against *E. coli* and *S. aureus*. Additionally, mechanical, morphological, and biodegradable characteristics were enhanced [60].

### 3.4. Wound Dressing and Healing

**Figure 21:**
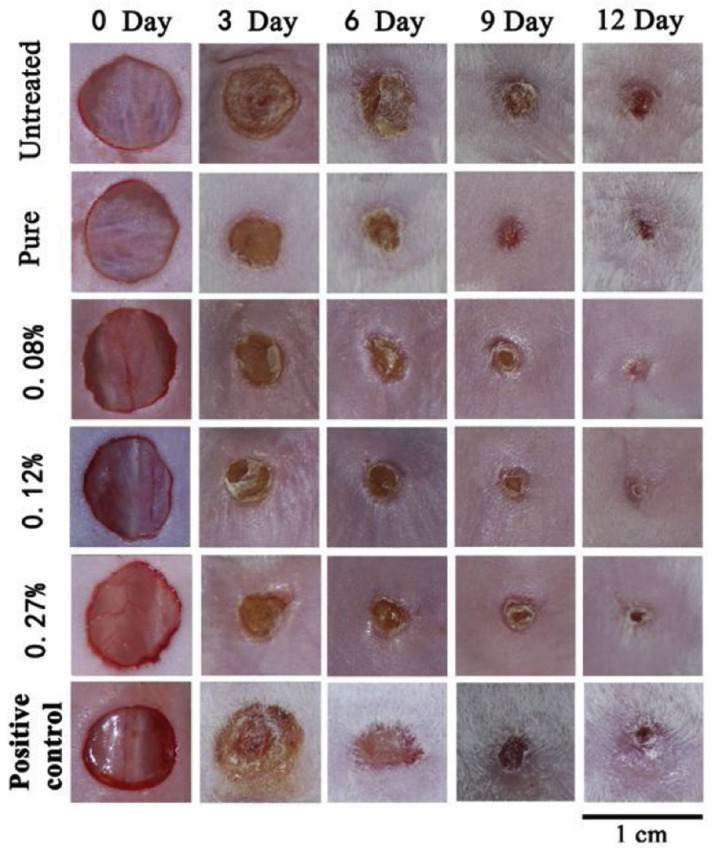
Stages of wound healing effects on mice with different levels of treated groups. Reprinted from [64]. Copyright 2020 with permission from Elsevier.

When combined with wound dressing materials and plasters, chitosan acts as an antibacterial agent against germs and viruses and provides the antibodies necessary for rapid wound healing. Incorporating silver nanoparticles, graphene oxide, chitosan, and curcumin into PVA nanofibers resulted in a hybrid composite. The antibacterial activity was shown to be superior to that of other nanoparticles, and the inclusion of graphene oxide boosts the mechanical characteristics. The in vitro test proved its biocompatibility, and it may be used to patch wounds that require both mechanical and antibacterial properties [61]. Chitosan coated with copper oxide and copper nanoparticles is produced for wound dressing applications. Copper oxide and copper nanoparticles with chitosan caps were synthesized via a simple chemical reduction of Cu2+ ions using ascorbic acid and sodium hydroxide. The antibacterial activity of the produced composite was evaluated using the inhibitory zone method against Gram-positive and -negative bacteria and found to be significantly greater than that of other nanomaterials, indicating that it is a superior alternative for wound dressing [62]. Electrospun polyaniline/chitosan nanofiber membranes were created and evaluated for their antibacterial activity in the treatment of chronic wounds and for their ability to minimize wound bioburden. The results suggested that increasing the concentration of polyaniline enhances the bactericidal activity and that it is also efficient against both Gram-positive and -negative bacteria. This property makes the polyaniline/chitosan membrane ideal for wound dressings and other healthcare applications [63]. For antimicrobial and wound healing applications, polyvinyl/chitosan nanofibers were combined with carboxymethyl chitosan nanoparticles encapsulated with an antibacterial peptide. These nanofibers containing varying concentrations of nanoparticles were applied to a mouse’s skin wound and demonstrated improved wound healing and antibacterial activity. The improved day wise wound healing progress in the mouse skin with different levels of concentration is shown in Figure 21 [64].

**Figure 22:**
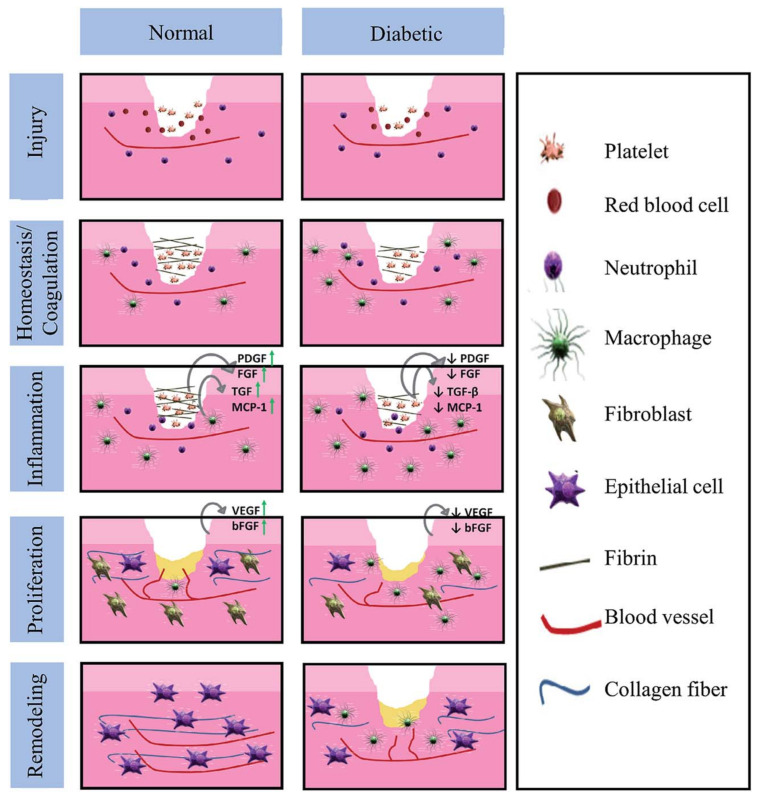
Differences in the wound healing phases between normal and diabetic persons. Reprinted from [65]. Copyright 2013 with permission from Elsevier.

There are many factors that affect the wound healing process. The process and time taken to heal a wound completely will vary between a normal and a person with diabetes. Factors such as infection, oxygenation, interruption of foreign bodies, wound depth and area, age, gender, obesity, medications, smoking, and alcoholism influence the wound healing process. Figure 22 indicates the various stages of the wound healing process. The wound healing process involves various stages such as coagulation, inflammation, proliferation, and remodeling [65].

### 3.5. Food Preservation and Packaging

Chitosan has the capacity to extend the shelf life of raw meat and food items while also minimizing bacterial and viral attack in preserved food. This enables naturally accessible fruits and vegetables to be preserved for longer lengths of time without the addition of preservatives. Chitosan was created in conjunction with zinc oxide coated in gallic acid films to provide an eco-friendly material for food packaging. The addition of gallic acid to chitosan increases its mechanical properties, and SEM images can be used demonstrate that the materials are compatible, suggesting that it could be used as an active material in food packaging [66]. Bacteria such as Escherichia coli and Salmonella enterica serovar typhimurium, which cause food contamination, have been researched using chitosan-based nanofibers. Chitosan nanofibers are created by electrospinning polyethylene oxide. Resistance to germs was determined in vitro, and the shelf life and preservation of red meat were also tested. The results demonstrated that the chitosan membrane was bactericidal, with a 99.9% decrease rate. The fresh meat’s shelf life was also increased by seven days, confirming chitosan’s contributions to the meat preservation and food packaging industries [67]. The antibacterial activity of chitosan-based nanofiber membranes generated via electrospinning was evaluated against Gram-positive and -negative bacteria. The results suggested that chitosan nanofibers operate as bacterial disruptors and perforators, as when the chitosan membrane comes into contact with negatively charged bacterial cells the membrane ruptures and protein and DNA leakage occur. As a result, it was established that chitosan membranes are good materials for food packing because they help prevent the spread of flora and infections [68].

**Figure 23:**
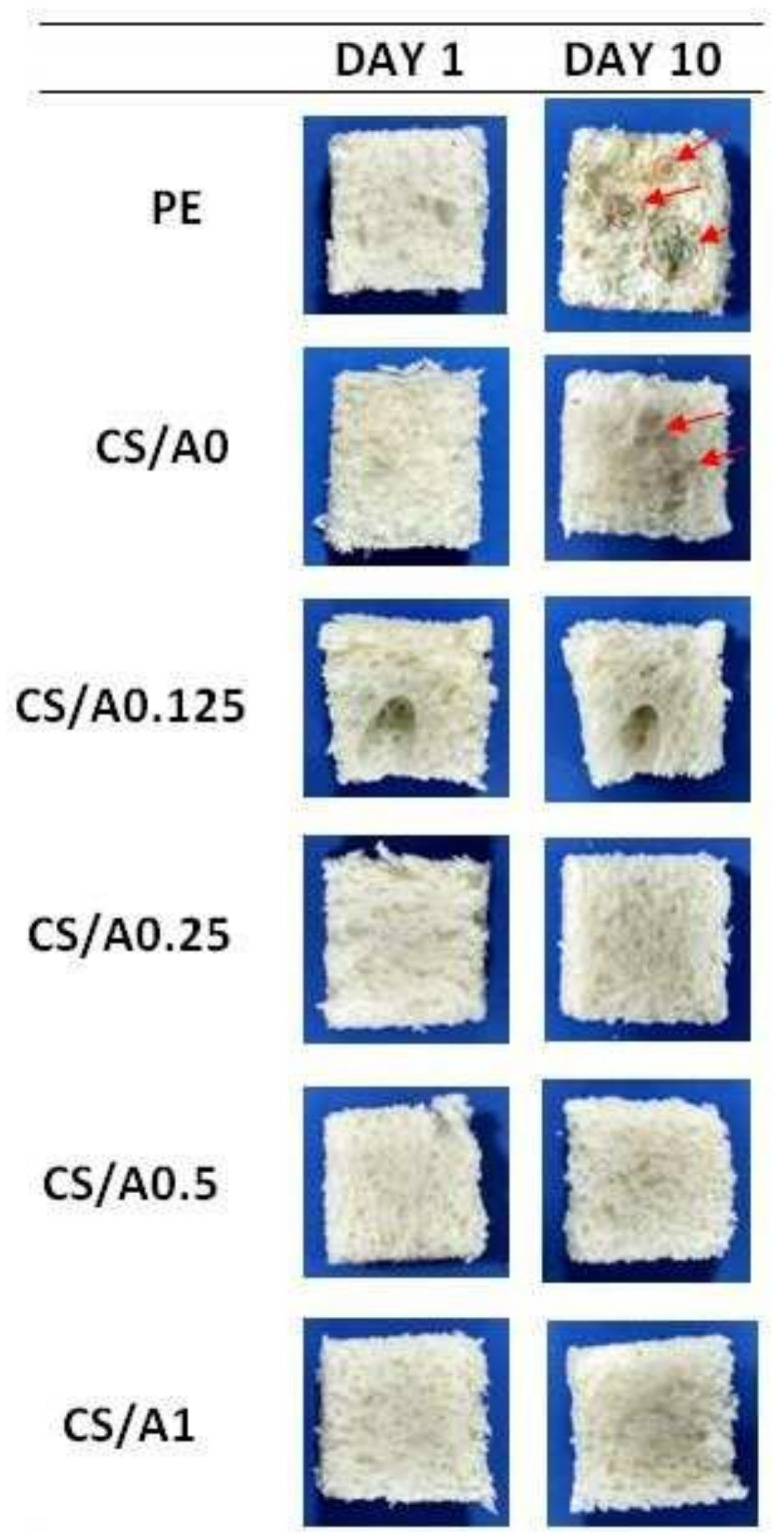
Inhibition of fungal growth on bread pieces packed in modified chitosan films (fungal growth is indicated in red arrows). Reprinted from [69]. Copyright 2018 with permission from Elsevier.

The fungal growth on the bread pieces packed in various treated packages was observed for 10 days and the results were recorded. It was found that bread pieces packed in LDPE and neat chitosan packages showed fungal growth after 10 days, but packages made of chitosan–AKEO films showed no fungal growth, meaning the antifungal properties were proven. The fungal growth levels on the bread pieces on day 10 with various levels of coating on the packaging material are shown in Figure 23 [69].

**Figure 24:**
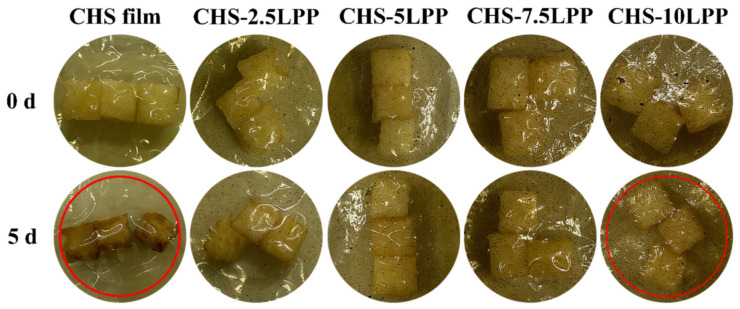
Fresh cut apples stored in chitosan and various concentration of LPP packages after 5 days [70].

The main reason for the weight loss in the organic substances is the dry matter consumption and stomatal transpiration due to respiration process. A weight loss of 28% was observed after 5 days of storage in chitosan film, which was found to be lower in films made using chitosan LPP groups. In particular, the package with chitosan and 10LPP showed lowest the weight loss of about 6.5%. The hydrogen bond generation between LPP and CS was the reason behind this result, as it reduces the water vapor loss from the package. Apple pieces packed in packages with different levels of chitosan LPP concentrations is shown in Figure 24 [70]. Organic foods and foods subjected to decay are stored in packaging materials embedded or reinforced with chitosan, so that the antimicrobial action of chitosan protects the food product from the attack of bacteria and viruses and extends the shelf life by retaining the moisture content in the food product itself. As the chitosan is non-toxic, if by any chance it mixes with the food items it will not be harmful to humans and will maintain the freshness of the food products. If chitosan is added to any kind of plastic used for packaging purposes, then after usage if the package is removed, the chitosan will take care of the biodegradation process.

### 3.6. Agricultural Activities

**Figure 25:**
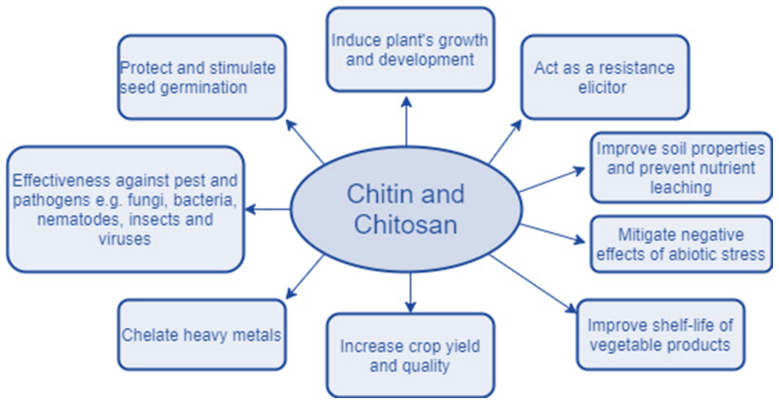
Possible applications of chitin and chitosan in agriculture [72].

The ability of fruits and leaves to retain moisture can be enhanced by covering them with chitosan. This also increases the shelf life of the fruits and vegetables. Chitosan cerium oxide nanoparticles were synthesized from spherical plant leaves and demonstrated superior antibacterial properties against infections, as well as being effective in biomedical applications. Figure 25 explains the effects of chitin and chitosan on plants [71]. 

To inhibit the growth of *S. aureus* and *E. coli*, chitosan-based coating films with varying titanium dioxide concentrations were produced. Chitosan with 0.05% titanium dioxide nanoparticles displayed the best thermal stability and exhibited superior inhibitory actions against bacteria; additionally, it was suggested that chitosan be utilized as a packaging material for vegetables and fruits to extend their shelf life [73]. To prevent blueberries from bacterial attack, they were preserved using chitosan/silica/nisin sheets. The results indicated that the pH value increased as a result of the addition of nisin, and that the turbidity level was also elevated. When nanoparticles are added to chitosan membranes, their tensile strength and ductility are decreased. The results demonstrated that the fruits lost some moisture and that CH-SN-N films can be utilized to preserve blueberries and extend their shelf life [74].

**Figure 26:**
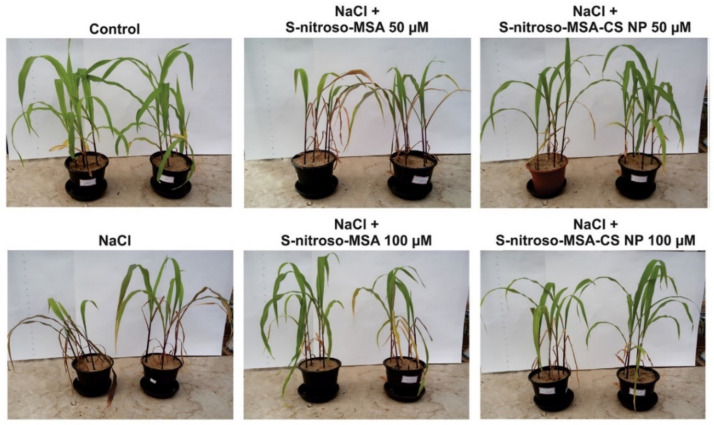
Plants under salt stress and under different treatments [75].

The maize plants under salt stress were taken for testing and treated with free S-nitroso-mercaptosuccinic acid and compared with another set of plants treated with the same acid encapsulated with chitosan nanoparticles at different concentrations. The 100 µM S-nitroso-MSA-chitosan treatment was found to provide effective relief against salt stress in the plants. The conditions of the plants with different levels of chitosan treatment in combination with NaCl are shown in Figure 26 [75].

**Figure 27:**
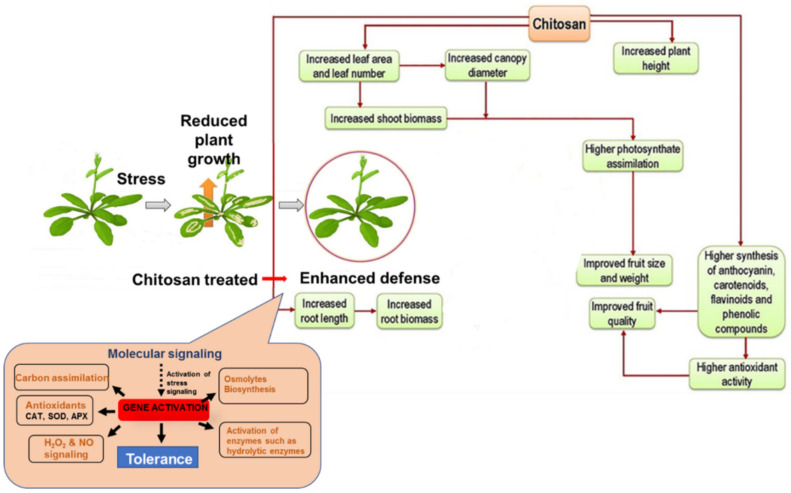
Plant growth conditions under the influence of chitosan [76].

Chitosan is a natural growth regulator that improves the defense against diseases. Chitosan improves plant growth by increasing water and nutrient intake. Chitosan helps in generating hydrolytic enzymes, which helps with the mobilization of starch and proteins. Plant hormones such as auxin and cytokinin were activated by chitosan, which promoted the root cells and increased nutrient intake. Seeds that underwent chitosan priming showed stimulated germination and vigor index rates. Genetic activation by chitosan in plants improves the growth of roots and the root biomass, meaning the canopy diameter, leaf area, and number of leaves, and height of the plant are increased. Due to the higher levels of photosynthesis, the fruit size and weight and the overall quality of the fruits are also improved. The influence of chitosan in the growth and quality of output from plants in the form of fruit is explained in Figure 27 [76]. The retention of moisture in fruits and vegetables after harvesting is ensured by coating them with chitosan. This keeps the fruit fresh and enhances the shelf life post-harvesting.

### 3.7. Drug Delivery

Chitosan has excellent pH sensitivity, and by combining it with hydrophobic groups, more flexible chitosan polymers are formed. Chitosan can be employed in cancer drug delivery because it can release doxorubicin, an anticancer agent, into tumor cells at a lower pH level, resulting in increased antitumor activity [77]. The most critical properties required of medicinal and pharmaceutical materials are biocompatibility and long-term stability. The carbon quantum dots were synthesized using chitosan. Quantum dots with carbon as their core exhibit visible-range luminescence and have been demonstrated to be useful in controlled medication delivery and cell labelling [78]. Curcumin–chitosan–zinc oxide was synthesized using a one-pot technique. It was discovered that it has higher influence against MRSA and *E. coli* than commercially supplied amoxicillin. Following an examination of the CCZ’s cytotoxic effects on grown human breast cancer cells, it was determined that it has potential for advanced medicinal applications [79]. For biomedical applications, bioinspired membranes comprised of green nanosilver and chitosan were created using a bottom-up eco-friendly design. This composite material demonstrated improved hemocompatibility, a high antioxidant capacity, and antiproliferative activity against cancer cells, as well as no toxicity against normal cells [80]. Graphene sheets coated in chitosan nanoparticles were tested against multidrug-resistant bacteria and found to be 90% harmful to Artemia franciscana after a 24 h incubation period [81]. Chitosan is frequently employed in the field of medical science due to its biodegradability and compatibility with living organisms. It is used to repair bone, regenerate tissue, and create dental adhesives, as well as to resist oral illnesses. Due to its special features, its use in dentistry is expanding [82]. Chitosan has been shown to be biocompatible, biosafe, and bioactive against the SARS, corona, and AIDS viruses, all of which pose a significant threat to human civilization. The addition of chitosan to ancient medications enhanced their antimicrobial properties [83].

**Figure 28:**
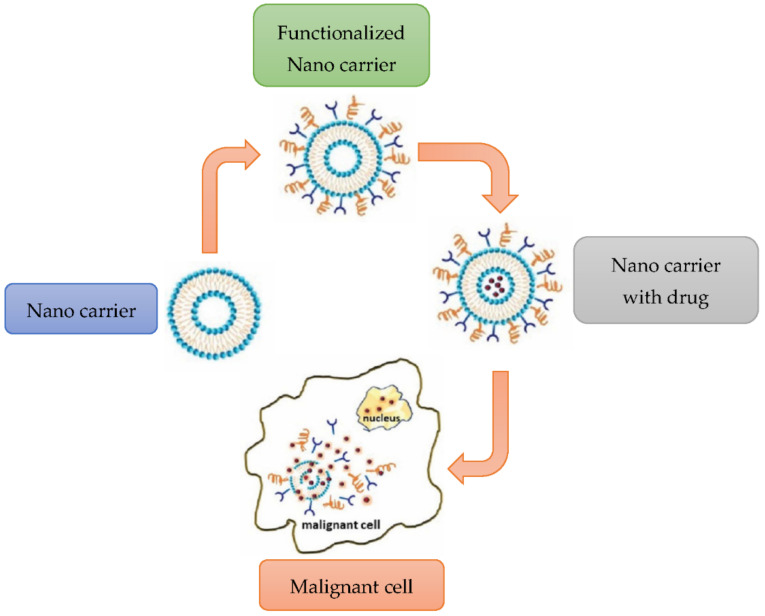
Process of drug delivery. Modified from [84].

Figure 28 shows the flow process through which a nanocarrier is converted into a functionalized nanocarrier that carries a drug and reacts with the malignant cell.

**Figure 29:**
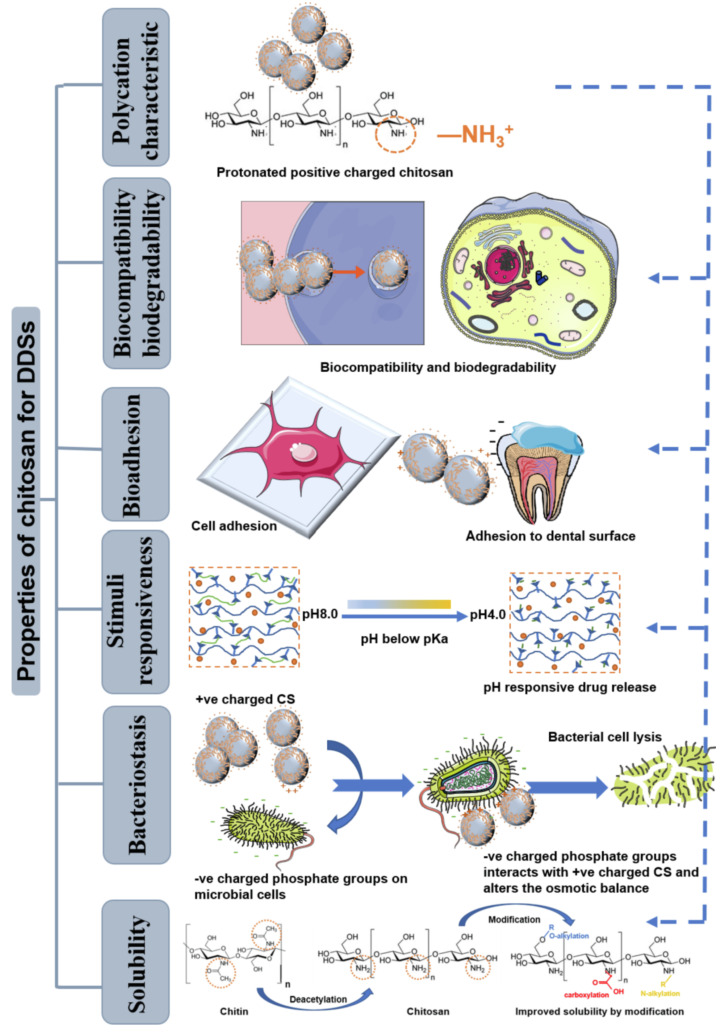
Characteristics and properties of chitosan as the best drug delivery agent. Reprinted from [85]. Copyright 2022 with permission from Elsevier.

The properties and characteristics related to drug delivery for chitosan are described in Figure 29. Polycation enhances absorption and mucoadhesion on dental surfaces, confirming the electrostatic interactions between negatively charged proteins and surfaces. When the drug delivery is increased, this increases the number of positive charges, which improves the mucoadhesion. Chitosan showed 15 to 40% weight reduction rates after 90 days of implantation. No allergy or toxicity was experienced during human trials due to the biocompatibility and biodegradability of chitosan. The adhesion of the drug to the surface must be ensured for a sufficient amount of time, and this was possible due to chitosan’s bioadhesion properties. The bacteriostasis properties of chitosan ensures the inhibition of bacteria and other microorganisms. Stimuli responsiveness allows the release of drugs based on changes in the environmental conditions, meaning such systems are termed “intelligent” drug delivery systems. Chitosan is soluble under acidic environments, and to improve the solubility and drug delivery ability, quaternization, carboxylation, and sulfation are performed [85]. Due to the abovementioned properties of chitosan, it is considered to be the best drug carrying and drug delivery agent.

### 3.8. Other Applications

Vacuum filtration was used to add low-viscosity chitosan to carrot cellulose nanofibrils in the range of 9 to 33% on a weight basis. The results indicated that adding chitosan improves the contact angle with water and renders the composite surface hydrophobic. The addition of chitosan increases the thermal stability and decreases the Young’s modulus, while the inhibitory effects increase with the increasing chitosan concentration [86]. The silver/chitosan nanoparticles were formed in situ and exhibited superior mechanical characteristics and stability in bodily fluid. The nanoparticles had antibacterial effects against *E. coli* and Staphylococcus aureus bacterial strains [87]. Natural chitosan and gelatin polymers synthesized in ternary solvents have a better water absorption capacity and are referred to as green superabsorbent polymers. Under optimum conditions, the composite reached water saturation in less than 60 min. Additionally, it has a high capacity for water absorption throughout broad temperature, pH, and salt concentration ranges. Without undergoing any chemical reactions, gelatin and chitosan can be mixed [88]. Chitosan/gold nanoparticles efficiently inhibit bacterial activity in human cells. To investigate the interaction of chitosan with bacterial membranes, simulation models were created. The antibacterial activity of the Cs-Au nanoparticles was determined to be satisfactory when compared to the simulated model [89]. The addition of rhamnolipids to chitosan was shown to be successful in developing a nanocomposite that targets Gram-positive bacteria. C/RL nanocomposites exhibit enhanced antibacterial activity while exhibiting less cytotoxicity, making them more suited for pharmaceutical applications [90]. To improve the tensile characteristics and hydrophobicity of chitosan, montmorillonite packed with carboxymethyl cellulose was added, resulting in good dispersion of nanoclay. Increased MMT addition disrupts the biopolymer plasticizer interactions, increasing the surface’s wettability [91]. OCMCS-SB, a stabilizer agent synthesized from chitosan and palladium, was tested for stability during Suzuki reactions and found to be satisfactory, with the possibility of further applications in organic transformations [92]. To increase the efficiency of the solar steam generator, semi-conductive in-situ-polymerized MnO2 nanowire–chitosan hydrogels were vertically stacked in macropore water channels. SPM-CH hydrogels enhance the lattice vibrations, while the polymeric network facilitates the creation of intermediate water clusters for steam generation. The solar energy conversion efficiency was determined to be 90.69% while the solar absorption was determined to be 94% using these hydrogels [93]. Separators for microbial fuel cells were built from self-assembled chitosan/montmorillonite. The resistance was lowered by 73.2%, increasing the proton conductivity, while the anode and cathode charge transfer impedances were reduced by 96.44 and 66.14%, respectively [94]. Pure chitosan was also used in the production of wine [95]. Chitosan can also be used as a biodegradable adhesive in woodworking. Synthetic adhesives are hazardous, unsustainable, and volatile. Chitosan-oxidized starch adhesives cure at lower temperatures and exhibit superior bonding and water resistance [96].

**Figure 30:**
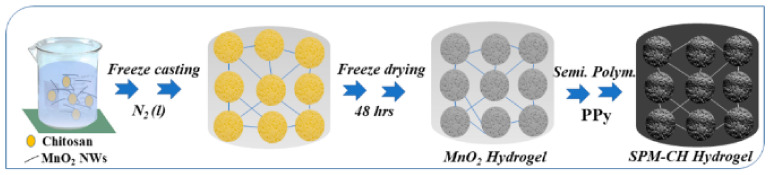
Process involved in making SPM–chitosan hydrogels. Reprinted from [93]. Copyright 2021 with permission from the American Chemical Society.

Manganese dioxide nanowires in chitosan solution were freeze-cast in the presence of nitrogen and then freeze-dried for 48 h, leading to the formation of MnO_2_ hydrogel. The semi-conductive in situ polymerization of polypyrrole films of SPM-CH hydrogel enhances the wettability, tortuosity, and solar absorption. The production process for the SPM–chitosan hydrogel is shown in Figure 30 [93]. The membranes used for the fuel cell are produced via a membrane casting process. The membranes are made of chitin nanowhiskers arranged in a chitosan matrix. Due to the higher proton conductivity and lower methanol permeability, there is great potential for use as electrolyte membranes in fuel cells [97]. Chitosan combined with silica on quartz crystal microbalance sensors provided better film formation abilities in the composite. The modified sensor showed improved sensitivity and reliability, and this type of sensors can be used to detect humidity in the air [98]. Table 4 indicates the various applications of chitin and chitosan and provides recommendations with respect to their capability for drug delivery, as well as their molecular weights.

## 4. Conclusions

This work has concentrated on the numerous applications of chitosan and its dominant traits, including its biocompatibility, antibiotic capabilities, and antibacterial activities. It is readily available as its principal source is the waste from marine species, and because its conversion requires simple chemical processes, the cost of the chitosan is low. It has vast and unique applications in medicine, food preservation and packaging, waste water filtration, dye removal from industrial effluents, wound healing, cancer cell treatment, air filtration, and bone replacements and implants, as well as to enhance the efficiency of solar cells. The water filtration membrane’s bioactivity and antifouling qualities are also enhanced. It has also been stated that chitosan has bioactive effects against SARS and COVID-19 viruses, which pose a threat to humans at the current time. Apart from the above-listed applications, chitosan is also used in wine making and is combined with natural adhesives to make it biodegradable. Chitosan also offers moderate mechanical capabilities with good surface hydrophilicity attributes.

## 5. Future Perspectives

Apart from bactericidal and biocompatibility uses, the material’s mechanical and thermal qualities must be enhanced when combined with other materials. Currently, it is employed in conjunction with other materials in water filtration membranes via coating and electrospinning methods. The temporal window within which the membrane bioactivity is most effective has not been well characterized. Chitosan is employed in bone replacements and dental implants due to its non-toxic and biocompatible nature; however, its mechanical qualities such as its strength, corrosion and wear resistance, and toughness are not well defined, which may provide potential opportunities in the development of biomaterials. The addition of chitosan to composite materials used in food packaging and preservation will also have a significant influence, as during natural disasters, food must be preserved for extended periods of time before being consumed, necessitating a longer shelf life. Moisturizing and retaining moisture on the skin’s surface is a difficult task in cosmetics. If chitosan is incorporated into cosmetics and lotions, it will work as an antibiotic and provide antimicrobial resistance against a variety of airborne bacteria and viruses that cause fatal infections. Additional studies must be conducted on waste water filtration to ensure that contaminants are removed efficiently and at a low cost. Incorporating or embedding chitosan into masks, textiles, gloves, and personnel breathing systems will require additional research, and innovative materials resistant to disease-causing substances will need to be developed. In some articles, it has been stated that chitosan can absorb fat from the foods we consume and that it also aids in weight loss. However, no work has shown these aspects empirically, and if established these advantages would represent significant advances in the field of medical science. Nano forms of chitosan can be obtained and filled along with other composites to study the differences in the properties. Catalysts must be developed for use in chitosan conversion processes [100]. Chitosan can be developed and used in dabs, suspensions, wipes, strands, frameworks, drinks, photography, and hydroponics [101].

**Figure 31:**
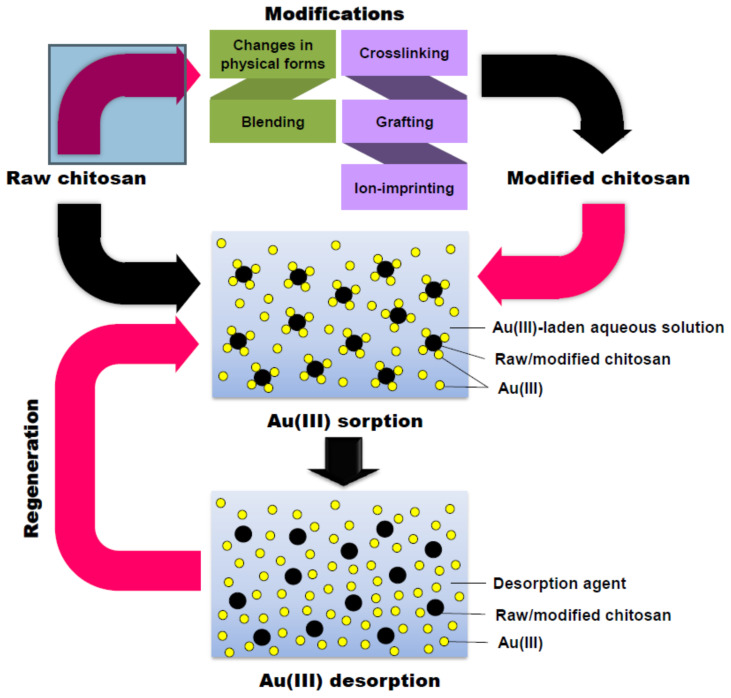
Process flow of gold recovery from aqueous solution using raw and modified chitosan. Reprinted from [102]. Copyright 2021 with permission from Elsevier.

The step-by-step process of converting raw chitosan into modified chitosan and the sorption, adsorption, and regeneration of chitosan are shown in Figure 31. Chitosan has applications in the recovery of gold from aqueous solutions via modification. Raw chitosan due to its Mw and degree of deacetylation affects the metal absorption ability, meaning the raw chitosan must be modified. Modifications were performed chemically and physically, and the modified chitosan showed better metal absorption and reusability abilities than raw chitosan [102]. Among the available formulations, chitosan-based nanocarriers are promising sources for the treatment of breast cancer and inclusion of chitosan, which when used as a drug carrier will bring about immense changes in the cancer treatment by reducing the cost and increasing the rates of survival and recovery [103]. In dental treatments, the usage of chitosan is also increasing due to its unique characteristics, such as its biocompatibility, biodegradability, hydrophilicity, antifungal activity, and bioactivity. In the future, chitosan will play a major role in dental repairs and in producing teeth with antimicrobial activity [82]. The incorporation of chitosan in medication such as in drug delivery will improve the treatment quality and the patient’s recovery rate from illness. Microneedles are under development in drug delivery and the increasing demand for their use will necessitate larger-scale production. The product design process is very important for microneedles if they are to be accepted by patients. Chitosan-based microneedles can be developed so that in future pandemics the diseases can be treated in an effective way [104]. Chitosan can also be used as a catalyst and in the trans-esterification of various oils in the presence of methanol. Furthermore, 90% biodiesel yield can be obtained using chitosan–cryogel beads over 8 to 32 h. In the future, chitosan in combination with other elements could be used for the extraction of biodiesels from various oils by acting as a catalyst. At the same time, it could be used in combination with biodiesel to investigate the pollution created so that newer processes can be implemented to increase efficiency. Chitosan is also used in the polymer electrolyte membrane in the fuel cells, as normal electrolyte fuel cells are expensive. The ionic conductivity can be improved via crosslinking, meaning cost-effective, biodegradable electrolyte membranes can be produced. In the future, the usage of electric and fuel-cell-operated vehicles will increase and the world will be in need of low-cost and ecofriendly electrolyte membranes with high levels of conversion efficiency. Chitosan will be the most suitable material for such purposes [105].

**Figure 32:**
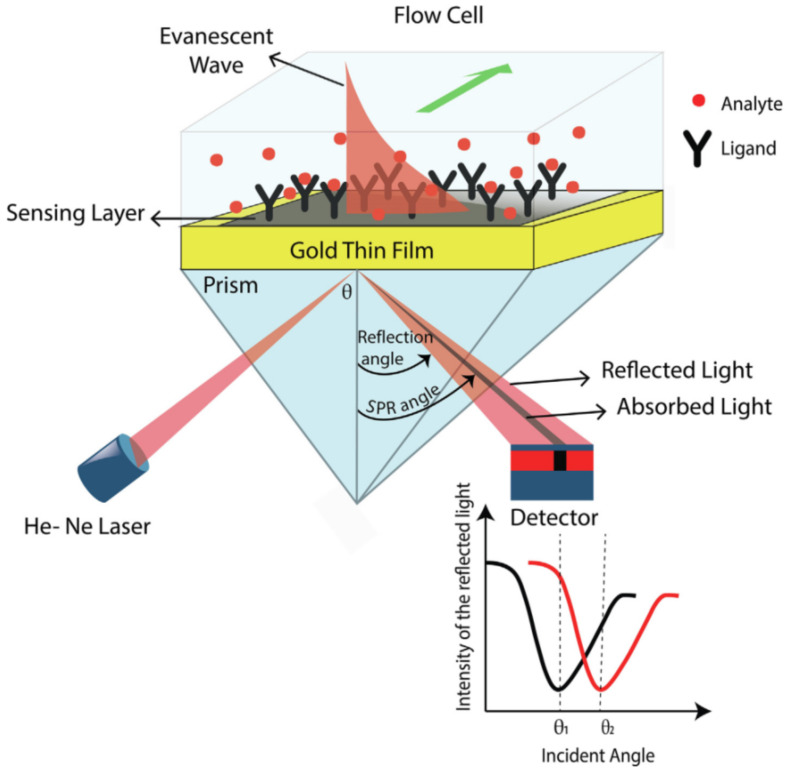
Surface plasmon resonance sensor setup. Reprinted from [106]. Copyright 2021 with permission from Elsevier.

Figure 32 explains the working principles of a surface plasmon resonance sensor and how the quantum dots of chitosan and graphene influence the sensitivity of the sensor. The surface-modified sensor was used for femtomolar detection and it was found that the modification of the sensor chip with chitosan–graphene quantum dots improved its sensitivity. Quantum dots of chitosan and graphene form a thin film that changes the refractive index, thereby shifting the resonance angle [106]. On the whole, chitosan has applications in almost every field, especially in developing fields, being used in fuel cells, electrolytic membranes for battery, sensors with good sensitivity, biodiesel development and extraction, agriculture, post-harvest processing, plant growth enhancement, for the removal of herbicides and pesticides from soil, for the removal of colors from dyes in the textile industry, for the removal of microbes and heavy metal and ions from industrial effluents, for the extraction and separation of gold from aqueous solution, for the removal of contaminants from drinking water and air, and for use in low-cost and effective drug delivery agents, in addition to aiding in wound healing, dentistry repairs, and scaffold development; it even acts as an effective drug delivery agent in the treatment of breast cancer and other chronic diseases. Chitosan has great potential to be the next material to be utilized in multifarious applications.

## Figures and Tables

**Table 1:** Various applications of chitosan [12].

Applications	Examples
Chitosan	
Biomedical and pharmaceutical materials	Treating burns, drug delivery systems, dental repair and treatment, surgical structures, artificial skin, lenses for eyes, dialysis of blood, artificial blood vessels, antitumor and antibiotic uses, accelerated wound healing.
Cosmetics	Hair and skin care products
Tissue engineering	Regeneration of bones and tissues, repair of scaffolds, regeneration of sulphate sponges in bone, development of artificial pancreas, diabetes treatment.
Agriculture	Food and seed coating, removal of pesticides and herbicides from soil and water, excellent film coating with antimicrobial activities, preservation of post harvested foods, enhancing plant growth, enhancing soil quality.
Food and feed additives	Food and beverage de-acidification, color stabilization in foods, lipid absorption reduction, extension of natural flavor, antioxidant and food preservation, controlling agent, stabilizing agent, thickening agent, additives in livestock and fish food, manufacture of dietary fibers.
Water engineering	Treatment of waste water, removal of heavy metals from water, removal of pesticides and ions from water, dye removal from water, removal of petroleum products from water, removal of dyes from effluents, color removal from textile waste waters.

**Table 2:** Chemical and biological properties of chitosan [12].

Chemical Properties	Biological Properties
Nitrogen content is enhanced	Biocompatible and biodegradable
High hydrophilicity and crystallinity due to structure	Non-toxic to humans
Powerful nucleophile and weak base	Combines with microbial cells quickly
Increases viscosity by forming hydrogen bonds	Regenerates the gum tissues
Has reactive groups for crosslinking and chemical activation	Stops bleeding
Insoluble in water and organic solvents	Enhances bone formation and repair
Soluble in acids	Inhibits the growth of fungi
Leads to salt formation with organic and inorganic acids	Inhibits the growth of tumor cells
Has chelating properties	Enhances birth control
Ionic conductivity	Acts as a cholesterol-reducing agent
Act as a polyelectrolyte in acidic conditions	Anticancer agent
Combines with negatively charged molecules	Act as a nervous depressant
Better adsorption and entrapment properties	Improves the immune response
Better separation and filtration abilities	Combines with mammalians
Ability to form films	Safe for water treatment

**Table 3:** Influence of Mw and DA on physicochemical and biological properties [13].

Physicochemical Properties			
S.No.	Properties	Degree of N-acetylation (DA)	Molecular Weight (MW)
1	Solubility	Indirectly proportional	N/A
2	Crystallinity	Directly proportional	N/A
3	Viscosity	Indirectly proportional	N/A
4	Biodegradability	Directly proportional	Indirectly proportional
5	Biocompatibility	Indirectly proportional	N/A
**Biological properties**			
6	Antimicrobial	Indirectly proportional	Directly proportional
7	Analgesic	Indirectly proportional	N/A
8	Anticholestemic	N/A	Indirectly proportional
9	Antioxidant	Indirectly proportional	Indirectly proportional
10	Hemostatic	Indirectly proportional	N/A
11	Mucoadhesion	Indirectly proportional	Directly proportional
12	Permeation enhancing effect	Indirectly proportional	Directly proportional
13	Antitumor	N/A	Indirectly proportional

**Table 4:** Various applications of chitin and chitosan and general recommendations [99].

Application	General Recommendations	
Healing of wounds	Chitosan preferred over chitin due to higher drug delivery capability	
DD systems	Higher drug delivery capability and higher Mw	
Repairing of Scaffolds	Good proliferation and structure Higher Mw results in prolonged biodegradation	
Enzyme immobilization	Adsorption	Chitin used for positively charged and neutral proteins Chitosan used for negatively charged proteins It possess higher drug delivery capability
	Covalent	Chitosan is used for immobilization at multipoints Chitin with higher DD or chitosan with lower DD is used for single-point immobilization
	Encapsulation	Chitosan has higher Mw, higher drug delivery rates, and better retention Chitosan-–alginate PECs possess medium Mw and have better stability under different conditions
Food preservative	Higher drug delivery Medium and lower Mw values	
Waste water treatment	Depending on pollutants and water conditions such as pH and ionic strength. Chitosan is preferred over chitin due to higher drug delivery ability and lower crystallinity	
Metal reduction	Chitosan’s characteristics decide the metal reduction rate, higher DD rate, and lower Mw results in the stabilization of nanoparticles	

## Data Availability

Not applicable.

## Abbreviations

SARS	Severe Acute Respiratory Syndrome
AIDS	Acquired immunodeficiency syndrome
Co/MCM	Chitosan–cobalt–silica
PVA-Co-PE	Poly(vinyl alcohol-co-ethylene)
Cr(IV)	Chromium–IV
Cu(II)	Cupric oxide
PVDF-S/MIL100-CS	Polyvinylidene fluoride metal organic framework chitosan
MWNT	Multiwalled nanotubes
PVA	Polyvinyl alcohol
PAN	Polyacrylonitrile
PEG	Polyethylene glycol
MWCNT	Multiwalled carbon nanotubes
*E. coli*	Escherichia coli
*S. aureus*	Staphylococcus aureus
Ni^2+^	Nickel cation with two positive charges
NaBH_4_	Sodium borohydride
FESEM	Field emission scanning electron microscope
SEM	Scanning electron microscope
EDAX	Energy-dispersive spectroscopy
Ni	Nickel
CS	Chitosan
FT-IR	Fourier transform infrared spectroscopy
ZnPc-CS	Zinc phthalocyanine chitosan
GSNO-PL/CS	No donor S-nitrosoglutathione-pluronic-chitosan
BC-CS	Bacterial cellulose–chitosan
BC-COS	Bacterial cellulose–chitooligosaccharide
Ag/CS/Silica	Silver–chitosan–silica
UV radiation	Ultraviolet radiation
K	Kelvin
Cu_2_O	Cuprous oxide
CuSO_4_	Copper sulphate
DNA	Deoxyribonucleic acid
CH-SN-N	Chitosan silica nanoparticles
MRSA	Methicillin-resistant staphylococcus aureus
CCZ	Curcumin chitosan zinc oxide
CS-Au	Chitosan gold nanoparticles
C/RL	Chitosan rhamnolipid
MMT	Montmorillonite
OCMCS-SB	O—Carboxymethyl chitosan Schiff base
MnO_2_	Manganese dioxide
SPM-CH	Semi-conductive in-situ-polymerized MnO2 nanowire–chitosan hydrogels.
DD	Degree of deacetylation
Mw	Molecular weight

## References

[1] Kassem A., Ayoub G.M., Malaeb L. (2019). Antibacterial Activity of Chitosan Nano-Composites and Carbon Nanotubes: A Review. Sci. Total Environ..

[2] Pervez M.N., Balakrishnan M., Hasan S.W., Choo K.H., Zhao Y., Cai Y., Zarra T., Belgiorno V., Naddeo V. (2020). A Critical Review on Nanomaterials Membrane Bioreactor (NMS-MBR) for Wastewater Treatment. npj Clean Water.

[3] Yadav M., Goswami P., Paritosh K., Kumar M., Pareek N., Vivekanand V. (2019). Seafood Waste: A Source for Preparation of Commercially Employable Chitin/Chitosan Materials. Bioresour. Bioprocess..

[4] Upadhyay U., Sreedhar I., Singh S.A., Patel C.M., Anitha K.L. (2021). Recent Advances in Heavy Metal Removal by Chitosan Based Adsorbents. Carbohydr. Polym..

[5] Kulkarni N., Shinde S.D., Jadhav G.S., Adsare D.R., Rao K., Kachhia M., Maingle M., Patil S.P., Arya N., Sahu B. (2021). Peptide-Chitosan Engineered Scaffolds for Biomedical Applications. Bioconjugate Chem..

[6] Negi H., Verma P., Singh R.K. (2021). A Comprehensive Review on the Applications of Functionalized Chitosan in Petroleum Industry. Carbohydr. Polym..

[7] Salama A. (2021). Recent Progress in Preparation and Applications of Chitosan/Calcium Phosphate Composite Materials. Int. J. Biol. Macromol..

[8] Jaber N., Al-Remawi M., Al-Akayleh F., Al-Muhtaseb N., Al-Adham I.S.I., Collier P.J. (2022). A Review of the Antiviral Activity of Chitosan, Including Patented Applications and Its Potential Use against COVID-19. J. Appl. Microbiol..

[9] Sharifianjazi F., Khaksar S., Esmaeilkhanian A., Bazli L., Eskandarinezhad S., Salahshour P., Sadeghi F., Rostamnia S., Vahdat S.M. (2022). Advancements in Fabrication and Application of Chitosan Composites in Implants and Dentistry: A Review. Biomolecules.

[10] Spoială A., Ilie C.I., Ficai D., Ficai A., Andronescu E. (2021). Chitosan-Based Nanocomposite Polymeric Membranes for Water Purification—A Review. Materials.

[11] Gao Y., Wu Y. (2022). Recent Advances of Chitosan-Based Nanoparticles for Biomedical and Biotechnological Applications. Int. J. Biol. Macromol..

[12] El Kady E. (2019). Chitin, Chitosan and Glucan, Properties and Applications. World J. Agric. Soil Sci..

[13] Lv S.H. (2016). High-Performance Superplasticizer Based on Chitosan. Biopolymers and Biotech Admixtures for Eco-Efficient Construction Materials.

[14] Kumirska J., Weinhold M.X., Thöming J., Stepnowski P. (2011). Biomedical Activity of Chitin/Chitosan Based Materials- Influence of Physicochemical Properties Apart from Molecular Weight and Degree of N-Acetylation. Polymers.

[15] Gatto M., Ochi D., Yoshida C.M.P., da Silva C.F. (2019). Study of Chitosan with Different Degrees of Acetylation as Cardboard Paper Coating. Carbohydr. Polym..

[16] Bansal V., Sharma P.K., Sharma N., Pal O.P., Malviya R. (2011). Applications of Chitosan and Chitosan Derivatives in Drug Delivery. Biol. Res..

[17] Ilyas R.A., Aisyah H.A., Nordin A.H., Ngadi N., Yusoff M., Zuhri M., Rizal M., Asyraf M., Sapuan S.M., Zainudin E.S. (2022). Natural-Fiber-Reinforced Chitosan, Chitosan Blends and Their Nanocomposites for Various Advanced Applications. Polymers.

[18] Qasim S.B., Zafar M.S., Najeeb S., Khurshid Z., Shah A.H., Husain S., Rehman I.U. (2018). Electrospinning of Chitosan-Based Solutions for Tissue Engineering and Regenerative Medicine. Int. J. Mol. Sci..

[19] Green-warren R.A., Bontoux L., Mcallister N.M., Kovacevich D.A., Kuznetsova C., Tenorio M., Lei L., Pelegri A.A., Jonathan P. (2022). Determining the Self-Limiting Electrospray Deposition Compositional Limits for Mechanically Tunable Polymer Composites. ACS Appl. Polym. Mater..

[20] Islam S., Jadhav A., Fang J., Arnold L., Wang L., Padhye R., Wang X., Lin T. (2011). Surface Deposition of Chitosan on Wool Substrate by Electrospraying. Adv. Mater. Res..

[21] Garg U., Chauhan S., Upendra N., Jain N. (2019). Current Advances in Chitosan Nanoparticles Based Drug Delivery and Targeting. Adv. Pharm. Bull..

[22] Shiekh K.A., Ngiwngam K., Tongdeesoontorn W. (2022). Polysaccharide-Based Active Coatings Incorporated with Bioactive Compounds for Reducing Postharvest Losses of Fresh Fruits. Coatings.

[23] Zhang J., Zeng L., Sun H., Zhang J., Chen S. (2017). Using Chitosan Combined Treatment with Citric Acid as Edible Coatings to Delay Postharvest Ripening Process and Maintain Tomato (Solanum Lycopersicon Mill) Quality. J. Food Nutr. Res..

[24] Fosso-Kankeu E., de Klerk C.M., van Aarde C., Waanders F., Phoku J. Antibacterial Activity of a Synthesized Chitosan-Silver Composite with Different Molecular Weights Chitosan against Gram-Positive and Gram-Negative Bacteria. Proceedings of the International Conference on Advances in Science, Engineering, Technology and Natural Resources (ICA-SETNR-16).

[25] Khan S.A., Khan S.B., Kamal T., Yasir M., Asiri A.M. (2016). Antibacterial Nanocomposites Based on Chitosan/Co-MCM as a Selective and Efficient Adsorbent for Organic Dyes. Int. J. Biol. Macromol..

[26] Liu K., Cheng P., Wang Y., Zhong W., Lu Z., Li M., Liu Q., Wang W., Zhu Q., Wang D. (2017). Concurrent Filtration and Inactivation of Bacteria Using Poly(Vinyl Alcohol-Co-Ethylene) Nanofibrous Membrane Facilely Modified Using Chitosan and Graphene Oxide. Environ. Sci. Nano.

[27] Kolangare I.M., Isloor A.M., Karim Z.A., Kulal A., Ismail A.F., Inamuddin, Asiri A.M. (2019). M. Antibiofouling Hollow-Fiber Membranes for Dye Rejection by Embedding Chitosan and Silver-Loaded Chitosan Nanoparticles. Environ. Chem. Lett..

[28] Bandara P.C., Nadres E.T., Rodrigues D.F. (2019). Use of Response Surface Methodology to Develop and Optimize the Composition of a Chitosan-Polyethyleneimine-Graphene Oxide Nanocomposite Membrane Coating to More Effectively Remove Cr(VI) and Cu(II) from Water. ACS Appl. Mater. Interfaces.

[29] Cho K.Y., Yoo C.H., Won Y.J., Hong D.Y., Chang J.S., Choi J.W., Lee J.H., Lee J.S. (2019). Surface-Concentrated Chitosan-Doped MIL-100(Fe) Nanofiller-Containing PVDF Composites for Enhanced Antibacterial Activity. Eur. Polym. J..

[30] Chaudhary M., Maiti A. (2020). Fe–Al–Mn@chitosan Based Metal Oxides Blended Cellulose Acetate Mixed Matrix Membrane for Fluoride Decontamination from Water: Removal Mechanisms and Antibacterial Behavior. J. Memb. Sci..

[31] Alshahrani A.A., Algamdi M.S., Alsohaimi I.H., Nghiem L.D., Tu K.L., Al-Rawajfeh A.E., in het Panhuis M. (2020). The Rejection of Mono- and Di-Valent Ions from Aquatic Environment by MWNT/Chitosan Buckypaper Composite Membranes: Influences of Chitosan Concentrations. Sep. Purif. Technol..

[32] Zhang L., Li L., Wang L., Nie J., Ma G. (2020). Multilayer Electrospun Nanofibrous Membranes with Antibacterial Property for Air Filtration. Appl. Surf. Sci..

[33] Makaremi M., Lim C.X., Pasbakhsh P., Lee S.M., Goh K.L., Chang H., Chan E.S. (2016). Electrospun Functionalized Polyacrylonitrile-Chitosan Bi-Layer Membranes for Water Filtration Applications. RSC Adv..

[34] Jabur A.R., Abbas L.K., Moosa S.A. (2016). Fabrication of Electrospun Chitosan/Nylon 6 Nanofibrous Membrane toward Metal Ions Removal and Antibacterial Effect. Adv. Mater. Sci. Eng..

[35] Mohraz M.H., Golbabaei F., Yu I.J., Mansournia M.A., Zadeh A.S., Dehghan S.F. (2019). Preparation and Optimization of Multifunctional Electrospun Polyurethane/Chitosan Nanofibers for Air Pollution Control Applications. Int. J. Environ. Sci. Technol..

[36] Liu H., Huang J., Mao J., Chen Z., Chen G., Lai Y. (2019). Transparent Antibacterial Nanofiber Air Filters with Highly Efficient Moisture Resistance for Sustainable Particulate Matter Capture. iScience.

[37] Khoerunnisa F., Rahmah W., Seng Ooi B., Dwihermiati E., Nashrah N., Fatimah S., Ko Y.G., Ng E.P., Khoerunnisa F. (2020). Chitosan/PEG/MWCNT/Iodine Composite Membrane with Enhanced Antibacterial Properties for Dye Wastewater Treatment. J. Environ. Chem. Eng..

[38] Nayl A.A., Abd-elhamid A.I., Awwad N.S., Abdelgawad M.A., Wu J., Mo X., Gomha S.M., Aly A.A., Bräse S. (2022). Review of the Recent Advances in Electrospun Nanofibers Applications in Water Purification. Polymers.

[39] Ahmad I., Kamal T., Khan S.B., Asiri A.M. (2016). An Efficient and Easily Retrievable Dip Catalyst Based on Silver Nanoparticles/Chitosan-Coated Cellulose Filter Paper. Cellulose.

[40] Kamal T., Khan S.B., Asiri A.M. (2016). Nickel Nanoparticles-Chitosan Composite Coated Cellulose Filter Paper: An Efficient and Easily Recoverable Dip-Catalyst for Pollutants Degradation. Environ. Pollut..

[41] Zhang A., Zhang Y., Pan G., Xu J., Yan H., Liu Y. (2017). In Situ Formation of Copper Nanoparticles in Carboxylated Chitosan Layer: Preparation and Characterization of Surface Modified TFC Membrane with Protein Fouling Resistance and Long-Lasting Antibacterial Properties. Sep. Purif. Technol..

[42] Bagheripour E., Moghadassi A.R., Hosseini S.M., Van der Bruggen B., Parvizian F. (2018). Novel Composite Graphene Oxide/Chitosan Nanoplates Incorporated into PES Based Nanofiltration Membrane: Chromium Removal and Antifouling Enhancement. J. Ind. Eng. Chem..

[43] Sangeetha K., Angelin Vinodhini P., Sudha P.N., Alsharani Faleh A., Sukumaran A. (2019). Novel Chitosan Based Thin Sheet Nanofiltration Membrane for Rejection of Heavy Metal Chromium. Int. J. Biol. Macromol..

[44] Vakili M., Deng S., Cagnetta G., Wang W., Meng P., Liu D., Yu G. (2019). Regeneration of Chitosan-Based Adsorbents Used in Heavy Metal Adsorption: A Review. Sep. Purif. Technol..

[45] Biao L., Tan S., Wang Y., Guo X., Fu Y., Xu F., Zu Y., Liu Z. (2017). Synthesis, Characterization and Antibacterial Study on the Chitosan-Functionalized Ag Nanoparticles. Mater. Sci. Eng. C.

[46] Venkatesan J., Anil S., Kim S.K., Shim M.S. (2017). Chitosan as a Vehicle for Growth Factor Delivery: Various Preparations and Their Applications in Bone Tissue Regeneration. Int. J. Biol. Macromol..

[47] Ali F., Khan S.B., Kamal T., Anwar Y., Alamry K.A., Asiri A.M. (2017). Anti-Bacterial Chitosan/Zinc Phthalocyanine Fibers Supported Metallic and Bimetallic Nanoparticles for the Removal of Organic Pollutants. Carbohydr. Polym..

[48] Cheah W.Y., Show P.L., Ng I.S., Lin G.Y., Chiu C.Y., Chang Y.K. (2019). Antibacterial Activity of Quaternized Chitosan Modified Nanofiber Membrane.

[49] Pelegrino M.T., Lima B.d.A., do Nascimento M.H.M., Lombello C.B., Brocchi M., Seabra A.B. (2018). Biocompatible and Antibacterial Nitric Oxide-Releasing Pluronic F-127/Chitosan Hydrogel for Topical Applications. Polymers.

[50] Yu X., Guo L., Liu M., Cao X., Shang S., Liu Z., Huang D., Cao Y., Cui F., Tian L. (2018). Callicarpa Nudiflora Loaded on Chitosan-Collagen/Organomontmorillonite Composite Membrane for Antibacterial Activity of Wound Dressing. Int. J. Biol. Macromol..

[51] Senthilkumar P., Yaswant G., Kavitha S., Chandramohan E., Kowsalya G., Vijay R., Sudhagar B., Kumar D.S.R.S. (2019). Preparation and Characterization of Hybrid Chitosan-Silver Nanoparticles (Chi-Ag NPs); A Potential Antibacterial Agent. Int. J. Biol. Macromol..

[52] Belbekhouche S., Bousserrhine N., Alphonse V., Le Floch F., Charif Mechiche Y., Menidjel I., Carbonnier B. (2019). Chitosan Based Self-Assembled Nanocapsules as Antibacterial Agent. Colloids Surf. B Biointerfaces.

[53] Yin N., Du R., Zhao F., Han Y., Zhou Z. (2020). Characterization of Antibacterial Bacterial Cellulose Composite Membranes Modified with Chitosan or Chitooligosaccharide. Carbohydr. Polym..

[54] Oh J.W., Chun S.C., Chandrasekaran M. (2019). Preparation and in Vitro Characterization of Chitosan Nanoparticles and Their Broad-Spectrum Antifungal Action Compared to Antibacterial Activities against Phytopathogens of Tomato. Agronomy.

[55] Zienkiewicz-Strzałka M., Deryło-Marczewska A., Skorik Y.A., Petrova V.A., Choma A., Komaniecka I. (2020). Silver Nanoparticles on Chitosan/Silica Nanofibers: Characterization and Antibacterial Activity. Int. J. Mol. Sci..

[56] Gadkari R.R., Ali S.W., Joshi M., Rajendran S., Das A., Alagirusamy R. (2020). Leveraging Antibacterial Efficacy of Silver Loaded Chitosan Nanoparticles on Layer-by-Layer Self-Assembled Coated Cotton Fabric. Int. J. Biol. Macromol..

[57] Jamshidi D., Sazegar M.R. (2020). Antibacterial Activity of a Novel Biocomposite Chitosan/Graphite Based on Zinc-Grafted Mesoporous Silica Nanoparticles. Int. J. Nanomed..

[58] Kızılkonca E., Torlak E., Erim F.B. (2021). Preparation and Characterization of Antibacterial Nano Cerium Oxide/Chitosan/Hydroxyethylcellulose/Polyethylene Glycol Composite Films. Int. J. Biol. Macromol..

[59] Zhou X., Yin A., Sheng J., Wang J., Chen H., Fang Y., Zhang K. (2021). In Situ Deposition of Nano Cu2O on Electrospun Chitosan Nanofibrous Scaffolds and Their Antimicrobial Properties. Int. J. Biol. Macromol..

[60] Babaee M., Garavand F., Rehman A., Jafarazadeh S., Amini E., Cacciotti I. (2022). Biodegradability, Physical, Mechanical and Antimicrobial Attributes of Starch Nanocomposites Containing Chitosan Nanoparticles. Int. J. Biol. Macromol..

[61] Dubey P., Gopinath P. (2016). PEGylated Graphene Oxide-Based Nanocomposite-Grafted Chitosan/Polyvinyl Alcohol Nanofiber as an Advanced Antibacterial Wound Dressing. RSC Adv..

[62] Jayaramudu T., Varaprasad K., Pyarasani R.D., Reddy K.K., Kumar K.D., Akbari-Fakhrabadi A., Mangalaraja R.V., Amalraj J. (2019). Chitosan Capped Copper Oxide/Copper Nanoparticles Encapsulated Microbial Resistant Nanocomposite Films. Int. J. Biol. Macromol..

[63] Moutsatsou P., Coopman K., Georgiadou S. (2018). Chitosan & Conductive PANI/Chitosan Composite Nanofibers—Evaluation of Antibacterial Properties. Curr. Nanomater..

[64] Zou P., Lee W.H., Gao Z., Qin D., Wang Y., Liu J., Sun T., Gao Y. (2020). Wound Dressing from Polyvinyl Alcohol/Chitosan Electrospun Fiber Membrane Loaded with OH-CATH30 Nanoparticles. Carbohydr. Polym..

[65] Moura L.I.F., Dias A.M.A., Carvalho E., de Sousa H.C. (2013). Recent Advances on the Development of Wound Dressings for Diabetic Foot Ulcer Treatment—A Review. Acta Biomater..

[66] Yadav S., Mehrotra G.K., Dutta P.K. (2021). Chitosan Based ZnO Nanoparticles Loaded Gallic-Acid Films for Active Food Packaging. Food Chem..

[67] Arkoun M., Daigle F., Heuzey M.C., Ajji A. (2017). Mechanism of Action of Electrospun Chitosan-Based Nanofibers against Meat Spoilage and Pathogenic Bacteria. Molecules.

[68] Arkoun M., Daigle F., Heuzey M.C., Ajji A. (2017). Antibacterial Electrospun Chitosan-Based Nanofibers: A Bacterial Membrane Perforator. Food Sci. Nutr..

[69] Priyadarshi R., Sauraj, Kumar B., Deeba F., Kulshreshtha A., Negi Y.S. (2018). Chitosan Films Incorporated with Apricot (Prunus Armeniaca) Kernel Essential Oil as Active Food Packaging Material. Food Hydrocoll..

[70] Jiang L., Luo Z., Liu H., Wang F., Li H., Gao H., Zhang H. (2021). Preparation and Characterization of Chitosan Films Containing Lychee (Litchi Chinensis Sonn.) Pericarp Powder and Their Application as Active Food Packaging. Foods.

[71] Senthilkumar R.P., Bhuvaneshwari V., Ranjithkumar R., Sathiyavimal S., Malayaman V., Chandarshekar B. (2017). Synthesis, Characterization and Antibacterial Activity of Hybrid Chitosan-Cerium Oxide Nanoparticles: As a Bionanomaterials. Int. J. Biol. Macromol..

[72] Shahrajabian M.H., Chaski C., Polyzos N., Tzortzakis N., Petropoulos S.A. (2021). Sustainable Agriculture Systems in Vegetable Production Using Chitin and Chitosan as Plant Biostimulants. Biomolecules.

[73] Xing Y., Li X., Guo X., Li W., Chen J., Liu Q., Xu Q., Wang Q., Yang H., Shui Y. (2020). Effects of Different Tio2 Nanoparticles Concentrations on the Physical and Antibacterial Activities of Chitosan-Based Coating Film. Nanomaterials.

[74] Sami R., Soltane S., Helal M. (2021). Microscopic Image Segmentation and Morphological Characterization of Novel Chitosan/Silica Nanoparticle/Nisin Films Using Antimicrobial Technique for Blueberry Preservation. Membranes.

[75] Bandara S., Du H., Carson L., Bradford D., Kommalapati R. (2020). Agricultural and Biomedical Applications of Chitosan-Based Nanomaterials. Nanomaterials.

[76] Chakraborty M., Hasanuzzaman M., Rahman M., Khan M.A.R., Bhowmik P., Mahmud N.U., Tanveer M., Islam T. (2020). Mechanism of Plant Growth Promotion and Disease Suppression by Chitosan Biopolymer. Agriculture.

[77] Tan L., Huang R., Li X., Liu S., Shen Y.M., Shao Z. (2017). Chitosan-Based Core-Shell Nanomaterials for PH-Triggered Release of Anticancer Drug and near-Infrared Bioimaging. Carbohydr. Polym..

[78] Janus Ł., Piatkowski M., Radwan-Pragłowska J., Bogdał D., Matysek D. (2019). Chitosan-Based Carbon Quantum Dots for Biomedical Applications: Synthesis and Characterization. Nanomaterials.

[79] Karthikeyan C., Varaprasad K., Akbari-Fakhrabadi A., Hameed A.S.H., Sadiku R. (2020). Biomolecule Chitosan, Curcumin and ZnO-Based Antibacterial Nanomaterial, via a One-Pot Process. Carbohydr. Polym..

[80] Barbinta-Patrascu M.E., Badea N., Bacalum M., Antohe S. (2020). Novel Bio-Friendly Nanomaterials Based on Artificial Cell Membranes, Chitosan and Silver Nanoparticles Phytogenerated From Eugenia Caryophyllata Buds: Eco-Synthesis, Characterization And Evaluation Of Bioactivities. Rom. Rep. Phys..

[81] Muthuchamy M., Govindan R., Shine K., Thangasamy V., Alharbi N.S., Thillaichidambaram M., Khaled J.M., Wen J.L., Alanzi K.F. (2020). Anti-Biofilm Investigation of Graphene/Chitosan Nanocomposites against Biofilm Producing P. Aeruginosa and K. Pneumoniae. Carbohydr. Polym..

[82] Zhang C., Hui D., Du C., Sun H., Peng W., Pu X., Li Z., Sun J., Zhou C. (2021). Preparation and Application of Chitosan Biomaterials in Dentistry. Int. J. Biol. Macromol..

[83] Meng Q., Sun Y., Cong H., Hu H., Xu F.J. (2021). An Overview of Chitosan and Its Application in Infectious Diseases. Drug Deliv. Transl. Res..

[84] Sharifi-Rad J., Quispe C., Butnariu M., Rotariu L.S., Sytar O., Sestito S., Rapposelli S., Akram M., Iqbal M., Krishna A. (2021). Chitosan Nanoparticles as a Promising Tool in Nanomedicine with Particular Emphasis on Oncological Treatment. Cancer Cell Int..

[85] Liu Z., Wang K., Peng X., Zhang L. (2022). Chitosan-Based Drug Delivery Systems: Current Strategic Design and Potential Application in Human Hard Tissue Repair. Eur. Polym. J..

[86] Szymańska-Chargot M., Chylińska M., Pertile G., Pieczywek P.M., Cieślak K.J., Zdunek A., Frąc M. (2019). Influence of Chitosan Addition on the Mechanical and Antibacterial Properties of Carrot Cellulose Nanofibre Film. Cellulose.

[87] Sun D., Turner J., Jiang N., Zhu S., Zhang L., Falzon B.G., McCoy C.P., Maguire P., Mariotti D., Sun D. (2020). Atmospheric Pressure Microplasma for Antibacterial Silver Nanoparticle/Chitosan Nanocomposites with Tailored Properties. Compos. Sci. Technol..

[88] Peng J., Wang X., Lou T. (2020). Preparation of Chitosan/Gelatin Composite Foam with Ternary Solvents of Dioxane/Acetic Acid/Water and Its Water Absorption Capacity. Polym. Bull..

[89] Fuster M.G., Montalbán M.G., Carissimi G., Lima B., Feresin G.E., Cano M., Giner-Casares J.J., López-Cascales J.J., Enriz R.D., Víllora G. (2020). Antibacterial Effect of Chitosan–Gold Nanoparticles and Computational Modeling of the Interaction between Chitosan and a Lipid Bilayer Model. Nanomaterials.

[90] Marangon C.A., Martins V.C.A., Ling M.H., Melo C.C., Plepis A.M.G., Meyer R.L., Nitschke M. (2020). Combination of Rhamnolipid and Chitosan in Nanoparticles Boosts Their Antimicrobial Efficacy. ACS Appl. Mater. Interfaces.

[91] Chen P., Xie F., Tang F., McNally T. (2021). Influence of Plasticiser Type and Nanoclay on the Properties of Chitosan-Based Materials. Eur. Polym. J..

[92] Dong Y., Bi J., Ming S., Zhang S., Zhu D., Meng D., Li T. (2021). Functionalized Chitosan as a Novel Support for Stabilizing Palladium in Suzuki Reactions. Carbohydr. Polym..

[93] Irshad M.S., Wang X., Abbasi M.S., Arshad N., Chen Z., Guo Z., Yu L., Qian J., You J., Mei T. (2021). Semiconductive, Flexible MnO2NWs/Chitosan Hydrogels for Efficient Solar Steam Generation. ACS Sustain. Chem. Eng..

[94] Yousefi V., Mohebbi-Kalhori D., Samimi A. (2020). Start-up Investigation of the Self-Assembled Chitosan/Montmorillonite Nanocomposite over the Ceramic Support as a Low-Cost Membrane for Microbial Fuel Cell Application. Int. J. Hydrogen Energy.

[95] Castro Marín A., Colangelo D., Lambri M., Riponi C., Chinnici F. (2021). Relevance and Perspectives of the Use of Chitosan in Winemaking: A Review. Crit. Rev. Food Sci. Nutr..

[96] Xi X., Pizzi A., Lei H., Zhang B., Chen X., Du G. (2022). Environmentally Friendly Chitosan Adhesives for Plywood Bonding. Int. J. Adhes. Adhes..

[97] Nasirinezhad M., Ghaffarian S.R., Tohidian M. (2021). Eco-Friendly Polyelectrolyte Nanocomposite Membranes Based on Chitosan and Sulfonated Chitin Nanowhiskers for Fuel Cell Applications. Iran. Polym. J..

[98] Qi P., Xu Z., Zhou T., Zhang T., Zhao H. (2021). Study on a Quartz Crystal Microbalance Sensor Based on Chitosan-Functionalized Mesoporous Silica for Humidity Detection. J. Colloid Interface Sci..

[99] Aranaz I., Mengibar M., Harris R., Panos I., Miralles B., Acosta N., Galed G., Heras A. (2009). Functional Characterization of Chitin and Chitosan. Curr. Chem. Biol..

[100] Chadha U., Selvaraj S.K., Ashokan H., Hariharan S.P., Mathew Paul V., Venkatarangan V., Paramasivam V. (2022). Complex Nanomaterials in Catalysis for Chemically Significant Applications: From Synthesis and Hydrocarbon Processing to Renewable Energy Applications. Adv. Mater. Sci. Eng..

[101] Chadha U., Bhardwaj P., Selvaraj S.K., Kumari K., Isaac T.S., Panjwani M., Kulkarni K., Mathew R.M., Satheesh A.M., Pal A. (2022). Advances in Chitosan Biopolymer Composite Materials: From Bioengineering, Wastewater Treatment to Agricultural Applications. Mater. Res. Express.

[102] Chang S.H. (2021). Gold(III) Recovery from Aqueous Solutions by Raw and Modified Chitosan: A Review. Carbohydr. Polym..

[103] Wang G., Li R., Parseh B., Du G. (2021). Prospects and Challenges of Anticancer Agents’ Delivery via Chitosan-Based Drug Carriers to Combat Breast Cancer: A Review. Carbohydr. Polym..

[104] Gorantla S., Dabholkar N., Sharma S., Rapalli V.K., Alexander A., Singhvi G. (2021). Chitosan-Based Microneedles as a Potential Platform for Drug Delivery through the Skin: Trends and Regulatory Aspects. Int. J. Biol. Macromol..

[105] Pal P., Pal A., Nakashima K., Yadav B.K. (2021). Applications of Chitosan in Environmental Remediation: A Review. Chemosphere.

[106] Kamal Eddin F.B., Fen Y.W., Omar N.A.S., Liew J.Y.C., Daniyal W.M.E.M.M. (2021). Femtomolar Detection of Dopamine Using Surface Plasmon Resonance Sensor Based on Chitosan/Graphene Quantum Dots Thin Film. Spectrochim. Acta—Part A Mol. Biomol. Spectrosc..

